# Differential regulatory T cell signature after recovery from mild COVID-19

**DOI:** 10.3389/fimmu.2023.1078922

**Published:** 2023-03-08

**Authors:** Pedro Henrique de Sousa Palmeira, Rephany Fonseca Peixoto, Bárbara Guimarães Csordas, Isac Almeida de Medeiros, Fátima de Lourdes Assunção Araújo de Azevedo, Robson Cavalcante Veras, Daniele Idalino Janebro, Ian P.G. Amaral, Tatjana Souza Lima Keesen

**Affiliations:** ^1^ Postgraduate program in Physiology Science, Immunology Laboratory of Infectious Diseases, Department of Cellular and Molecular Biology, Federal University of Paraiba, João Pessoa, Paraíba, Brazil; ^2^ Postgraduate program in Natural and Synthetic Bioactive Products, Immunology Laboratory of Infectious Diseases, Federal University of Paraiba, João Pessoa, Paraíba, Brazil; ^3^ Research Institute for Drugs and Medicines, Federal University of Paraiba, João Pessoa, Paraíba, Brazil; ^4^ Biotechnology Graduation Program, Immunology Laboratory of Infectious Diseases, Federal University of Paraiba, João Pessoa, Paraíba, Brazil; ^5^ Immunology Laboratory of Infectious Diseases, Department of Cellular and Molecular Biology, Federal University of Paraiba, João Pessoa, Paraíba, Brazil

**Keywords:** regulatory T cells, mild symptoms, immunomodulation, SARS-CoV-2, immune response, recovered COVID-19

## Abstract

Severe acute respiratory syndrome coronavirus 2 (SARS-CoV-2) is characterized by a range of symptoms in which host immune response have been associated with disease progression. However, the putative role of regulatory T cells (Tregs) in determining COVID-19 outcomes has not been thoroughly investigated. Here, we compared peripheral Tregs between volunteers not previously infected with SARS-CoV-2 (healthy control [HC]) and volunteers who recovered from mild (Mild Recovered) and severe (Severe Recovered) COVID-19. Peripheral blood mononuclear cells (PBMC) were stimulated with SARS-CoV-2 synthetic peptides (Pool Spike CoV-2 and Pool CoV-2) or staphylococcal enterotoxin B (SEB). Results of a multicolor flow cytometric assay showed higher Treg frequency and expression of IL-10, IL-17, perforin, granzyme B, PD-1, and CD39/CD73 co-expression in Treg among the PBMC from the Mild Recovered group than in the Severe Recovered or HC groups for certain SARS-CoV-2 related stimulus. Moreover, Mild Recovered unstimulated samples presented a higher Tregs frequency and expression of IL-10 and granzyme B than did that of HC. Compared with Pool CoV-2 stimuli, Pool Spike CoV-2 reduced IL-10 expression and improved PD-1 expression in Tregs from volunteers in the Mild Recovered group. Interestingly, Pool Spike CoV-2 elicited a decrease in Treg IL-17^+^ frequency in the Severe Recovered group. In HC, the expression of latency-associated peptide (LAP) and cytotoxic granule co-expression by Tregs was higher in Pool CoV-2 stimulated samples. While Pool Spike CoV-2 stimulation reduced the frequency of IL-10^+^ and CTLA-4^+^ Tregs in PBMC from volunteers in the Mild Recovered group who had not experienced certain symptoms, higher levels of perforin and perforin^+^granzyme B^+^ co-expression by Tregs were found in the Mild Recovered group in volunteers who had experienced dyspnea. Finally, we found differential expression of CD39 and CD73 among volunteers in the Mild Recovered group between those who had and had not experienced musculoskeletal pain. Collectively, our study suggests that changes in the immunosuppressive repertoire of Tregs can influence the development of a distinct COVID-19 clinical profile, revealing that a possible modulation of Tregs exists among volunteers of the Mild Recovered group between those who did and did not develop certain symptoms, leading to mild disease.

## 1 Introduction

In December 2019, pneumonia of unknown etiology was observed in Wuhan, China, and was later associated with a new betacoronavirus (1). Named Coronavirus of Severe Acute Respiratory Syndrome 2 (SARS-CoV-2), this new pathogen is the causative agent of Coronavirus Disease 2019 (COVID-19) and is responsible for the current pandemic announced by the World Health Organization (WHO) on 11 March 2020 (1–3). Although the disease may present with a broad spectrum of manifestations, including asymptomatic cases and severe symptoms that may result in death, most affected individuals develop a non-severe condition (3, 4).

The public health emergency caused by the SARS-CoV-2 infection has prompted the scientific community to evaluate immune response, revealing the significant contribution of the immune system to disease progression (5). In this context, factors such as viral load and the efficacy of innate immunity, especially those mediated by type I interferons, seem to be pivotal to adaptive responsiveness and clinical outcomes (6). For example, the time of action of CD4^+^ T lymphocytes has been associated with different clinical conditions (7). Additionally, lymphopenia and defects in Th1 immune profile have been associated with increased severity in COVID-19 cases (8).

Regulatory T cells (Tregs), a CD4^+^ T lymphocyte subpopulation with regulatory functions, develop a pivotal role in self-tolerance and immune homeostasis in some diseases (9, 10). In the context of COVID-19, alterations in IL-10^+^ and CD39^+^ Treg subpopulations or an imbalance between Tregs/Th17 can be associated with disease severity (10–13). However, divergent results regarding the frequency of these lymphocytes raise questions regarding the role of Tregs in disease progression (10, 14–16).

Thus, to better understand the role of Tregs in the context of SARS-CoV-2 infection, we evaluated Tregs (CD3^+^CD4^+^CD25^+^FOXP3^+^) based on their frequency and expression of cytokines, cytotoxic granules, inhibitory receptors, and ectonucleotidases in the peripheral blood mononuclear cells (PBMC) from volunteers recovered from mild and severe forms of COVID-19. Among the volunteers who recovered from mild disease, we compared the immune parameters between those who did and did not experience certain symptoms. We observed a differential Treg immune profile after disease resolution particularly in the group of volunteers who recovered from mild SARS-CoV-2 infection. This study points to a pivotal change in the Treg profile that could help the scientific community understand the role of this subpopulation of lymphocytes in the course of different clinical forms of COVID-19.

## 2 Materials and methods

### 2.1 Ethics statement

This study was approved by the National Commission of Ethics in Research (certificate CAAE: 31354720.0.0000.5188). All experiments were performed in compliance with the relevant regulations, institutional guidelines, and ethical standards of the Declaration of Helsinki. Informed consent was obtained from all the enrolled volunteers.

### 2.2 Patient recruitment

This study was conducted from May 2020 to May 2021 in Brazil. The study began around the time when the SARS-CoV-2 virus of the original lineage was still circulating and continued with the alpha and gamma variants being first detected in the end of 2020 (between October and December). These studies ended in May 2021, a few days after the delta variant was identified for the first time in Brazil (April 26, 2021) (17, 18). Sixteen nonvaccinated volunteers who presented COVID-19 symptoms between May 2020 and May 2021 and had subsequently recovered were recruited. The SARS-CoV-2 diagnosis was based on RT qPCR for COVID-19 during the acute phase and IgG serological tests (Euroimmun Anti‐SARS‐CoV‐2 assay, Perkin Elmer Company) after recovery. The recovered COVID-19 volunteers were assigned to either the Mild Recovered (did not need hospitalization, n = 9) or Severe Recovered (presented pulmonary symptoms requiring hospitalization, n = 7) group. For epidemiologic purposes, severe dyspnea, a respiratory rate of 30 or more breaths per minute, a blood oxygen saturation of 93% or less, and infiltrates in more than 50% of the lung were considered severe symptoms of COVID-19 (19). In addition, a healthy control group (HC, n = 8) was included. Volunteers in the HC group were not previously vaccinated against COVID-19, were reportedly asymptomatic for the last 10 weeks, were negative by certified SARS‐CoV‐2 antibody test (Euroimmun Anti‐SARS‐CoV‐2 assay Perkin Elmer Company), and had a negative RT qPCR test for SARS‐CoV‐2. Whole blood for flow cytometry analysis was collected in sodium heparin-coated vacutainers and gently agitated until processing. The blood samples were processed on the day of collection.

### 2.3 RT qPCR assay

Quantitative real-time PCR (RT qPCR) was performed as described previously (20) at the time of sample collection for flow cytometry experiments. This procedure was essential to ensure that no SARS-CoV-2 infection or reinfection occurred in the HC and recovered volunteers. The RNA isolated from nasopharyngeal or oropharyngeal swabs was extracted (QIAprep&amp Viral RNA UM Kit, QIAGEN, USA) and amplified by one-step RT qPCR (SARS-CoV-2 N1+N2 Assay Kits, QIAGEN, USA).

### 2.4 Design and preparation of SARS-CoV-2 epitope pools

For peptide design, we identify the most frequent HLA-class I/II alleles among the Brazilian population and most frequently in the world in the IEDB tools. We then performed MHC-I and II binding affinity and antigenicity predictions and analyzed the peptide molecular dynamics of the best-fitted MHC-I and II/protein of SARS-CoV-2 complexes. SARS-CoV-2 virus-specific CD4 and CD8 peptides (**Table 1**) **(**patent number: BR 10 2022 005518 1) were synthesized (GenOne Biotechnologies, Rio de Janeiro, Brazil), lyophilized, and stored at -20°C for subsequent analysis. Subsequently, the peptides were resuspended in H_2_O (MiliQ) and pooled into groups (Pool CoV-2 contained peptides from the spike protein and non-spike proteins, whereas Pool Spike CoV-2 contained peptides from the spike protein only, **Table 1**).

**Table 1:** List of peptides chosen in this study through prediction studies and Spike peptide pool.

		Name	Peptide	a.a.* position	Source
Pool CoV-2		ORF1a	ILASFSASTSAFVET	476-490	This study
		ORF1a	FLHFLPRVFSAVGNICYTP	2880-2890	This study
		ORF1b	FVDGVPFVVSTGYHFR	4726-4741	This study
	Pool Spike CoV-2	SPIKE_NTD	NIDGYFKIYSKHTPINLV	196-210	(21)
		SPIKE_RBD	ATRFASVYAWNRKRI	344-358	(21)
		SPIKE_S2	ALQIPFAMQMAYRFN	893-907	(21)
		ORF3	KKRWQLALSKGVHFV	66-80	This study
		E	FYVYSRVKNLNSSRV	56-70	This study
		M	LYLYALVYFLQSINFVRIIM	114-123	This study
		M	KEITVATSRTLSYYK	166-180	This study
		ORF8	FYSKWYIRVGARKSA	41-55	This study

*a.a, amino acid.

SARS-CoV-2 epitopes were predicted using protein sequences derived from the Brazilian SARS-CoV-2 sequences deposited in GenBank and IEDB analysis resources. CD4 SARS-CoV-2 epitope prediction was performed as previously reported, using the NetMHCIIpan 4.0 algorithm and CD4 immunogenicity prediction (IEDB), HLA-DRB*1:03:01, HLA-DRB*1:07:01, HLA-DRB*1:15:01, HLA-DRB*3:01:01, HLA-DRB*3:02:02, HLA-DR*4:01:01, and HLA-DRB*5:01:01. Due to extremely high heterogenicity of the Brazilian population, we carefully included the frequently identified HLA I and II in the Brazilian population along with the most frequently identified HLA populations in the world. CD8 SARS-CoV-2 epitope prediction was performed as previously reported using the NetMHCpan4.0 algorithm (HLA-A* 01: 01; HLA-A* 02: 01; HLA-A* 11: 01; HLA-A* 24: 02; HLA-A* 68: 01; HLA-A* 23: 01; HLA-A* 26: 01; HLA-A* 30: 02; HLA-A* 31: 01; HLA-B* 07: 02; HLA-B* 51: 01; HLA-B* 35: 01; HLA-B* 44: 02; HLA-B* 35: 03; HLA-C* 05: 01; HLA- C* 07: 01 e HLA-C* 15: 02). Despite inferring cellular populations of T lymphocytes, this strategy extrapolates to lymphocyte-like cells, such as natural killer (NK) cells, because a specific prediction program for NK cell populations is not yet available.

### 2.5 Isolation of plasma

Plasma samples were collected after centrifugation of whole blood at 400 × *g* for 10 min at room temperature (25°C). The undiluted plasma was then transferred to cryotubes, aliquoted, and stored at -80°C for subsequent analysis.

### 2.6 Isolation of PBMC

PBMC from volunteers inserted in the HC and volunteers who had recovered from mild and severe COVID-19 (Mild Recovered and Severe Recovered groups, respectively) were obtained from heparinized venous blood using density gradient centrifugation (Ficoll-Paque ™ Plus, GE Healthcare, Life Sciences, Pittsburgh, PA, USA). PBMC were centrifuged for 40 min at 400 × *g* and washed three times with phosphate-buffered saline (PBS) before counting. PBMC were maintained in RPMI-1640 (Sigma-Aldrich) supplemented with antibiotics (penicillin, 200 U/mL; streptomycin, 0.1 mg/mL), 1 mM L-glutamine (1 mM), and 10% heat-inactivated AB Rh+ human serum (Sigma-Aldrich), hereafter referred to as medium. Cultures were set up at a concentration of 2.5 X 10^5^ cells in 96-well plates in the presence or absence of SARS-CoV-2 antigens and Staphylococcal enterotoxin B from *Staphylococcus aureus* (SEB, Sigma-Aldrich). PBMC were subjected to four different conditions: unstimulated (medium), stimulated with SARS-CoV-2 antigens (Pool Spike Cov-2 and Pool Cov-2, each at 1 µg/well), and stimulated with SEB (1 ug/well). The cells were incubated under 5% CO_2_ in an incubator at 37°C for 16 h. Next, brefeldin-A (1 mg/mL, Sigma-Aldrich) was added and the samples were incubated under 5% CO_2_ in an incubator at 37°C for 4 h.

### 2.7 Flow cytometry assay

Briefly, freshly isolated PBMC were plated at a concentration of 2.5 x 10^5^ cells per well in a 96-well U-bottom plate. Cocktails of Antibodies for extracellular staining were added and incubated for 30 min at 4°C. For extracellular staining, the following antibodies from BD Biosciences were used: extracellular PE-Cy5 conjugated anti-CD3 (clone UCHT1, isotype. Mouse BALB/c IgG1, κ, cat. 555334, specie. mouse), APC-Cy7 conjugated anti-CD4 (clone RPA-T4, isotype. Mouse IgG1, κ, cat. 557871, specie. mouse), PE-Cy7 conjugated anti-CD25 (clone M-A251, isotype. Mouse BALB/c IgG1, κ, cat. 557741, specie. mouse), PE-conjugated anti-CD39 (clone TU66, Isotype: Mouse IgG2b, κ, cat. 555464, specie. mouse), APC-conjugated anti-CD73 (clone AD2, isotype: Mouse IgG1, κ, cat. 560847, specie. mouse), PE-conjugated anti-PD1 (clone MIH4, isotype. Mouse IgG1, κ, cat. 557946, specie. mouse), PE-conjugated anti-LAP (clone TW4-2F8, isotype. Mouse BALB/c IgG1, κ, cat. 562260, specie. mouse), IgG isotypes control antibodies such as IgG3-FITC (clone J606, isotype. Mouse BALB/c IgG3, κ, cat. 555578, specie. mouse), IgG3-Alexa Fluor 647 (clone J606, isotype. Mouse BALB/c IgG3, κ, cat. 560803, specie. mouse), IgG1-APC-H7 (clone MOPC-21, isotype. Mouse IgG1, κ, cat. 560167, specie. mouse), IgG1-PE-Cy-5 (clone MOPC-21, isotype. Mouse IgG1, κ, cat. 555750, specie. mouse), and IgG1-PE-Cy-7 (clone MOPC-21, isotype. Mouse IgG1, κ, cat. 557872, specie. mouse). After incubation with antibodies for 30 min at 4°C, the cells were washed with 150 μL of PBS. The plate was centrifuged (8 min, 244 × *g*, 4°C), the supernatant was removed, and 100 μL of 4% formaldehyde and 100 μL of PBS were added to the wells. The plate was incubated at room temperature (25°C) for 20 min to fix the extracellular staining. After centrifugation (8 min, 244 × *g*, 4°C), the supernatant was discarded and the samples were washed with 150 μL of PBS. The plate was centrifuged yet again (8 min, 244 × *g*, 4°C) and the supernatant was discarded. For intracellular staining, the cells were permeabilized with 150 μL of permeabilization buffer (0.5% bovine serum albumin (BSA), w/v and 0.5% saponin, w/v in PBS) for 10 min at room temperature (25°C). After centrifugation (8 min, 244 × *g*, 4°C), the supernatant was removed, and intracellular staining was performed. The following conjugated antibodies from BD Biosciences were used to detect intracellular molecules: Alexa Fluor 647 conjugated anti-perforin (clone δG9, isotype. Mouse BALB/c IgG2b, κ, cat. 563576, specie. mouse), PE-conjugated anti-granzyme B (clone GB11, isotype. Mouse BALB/c IgG1, κ, cat. 561142, specie. mouse), PE-conjugated anti-CTLA-4 (clone BNI3, isotype. Mouse BALB/c IgG2a, κ, cat. 555853, specie. mouse), APC-conjugated anti-IL10 (clone JES3-19F1, isotype. Rat IgG2a, κ, cat. 554707, specie. rat), PE-conjugated anti-IL-17 (clone SCPL1362, isotype. Mouse IgG1, κ, cat. 560436, specie. mouse), Alexa Fluor 488 conjugated anti-FOXP3 (clone 236A/E7, isotype. Mouse BALB/c IgG1, κ, cat. 561181, specie. mouse), and IgG isotype control antibodies IgG1-PE (clone MOPC-21, isotype. Mouse IgG1, κ, cat. 559320, specie. mouse). All antibodies were used according to the manufacturer’s instructions. The plate was then incubated for 45 min at room temperature (25°C), and 150 μL of permeabilization buffer was added. After centrifugation, the supernatant was removed (8 min, 244 × *g*, 4°C). Finally, 200 μL of wash B (PBS/BSA) was added, and the samples were transferred to fluorescence-activated cell sorting (FACS) tubes and maintained at 4°C. At least 70,000 gated events were acquired using FACS CANTO II (BD Biosciences) and analyzed using the FlowJo v.10.8 software (BD, Ashland - USA).

### 2.8 Flow cytometry data analysis

All parameters evaluated in Tregs were analyzed using FlowJo software v.10.8 (BD, Ashland, USA). Limits for quadrant markers were set based on negative populations (cells), isotype controls, and fluorescence minus one (FMO), when appropriate. Five or six different fluorochromes were identified in each analysis. Four fluorochromes, anti-CD3 PE-Cy5, anti-CD4 APC-Cy7, anti-CD25 PE-Cy7, and anti-FOXP3 Alexa Fluor 488, were used to identify Tregs (CD3^+^CD4^+^CD25^+^FOXP3^+^). We first selected the total lymphocyte gate through size-FSC-A and granularity-SSC-A profiles, followed by singlet separation using the FSC-A × FSC-H parameters. Next, we set the CD3^+^CD4^+^ cells and identified CD25 + T cells within this subpopulation. Subsequently, CD3^+^CD4^+^CD25^+^FOXP3^+^ T-cells were detected. Two fluorochromes, anti-X PE and anti-Y APC (or Alexa Fluor 647), were used to assess the intracellular or surface markers. The following markers were used: IL-10, IL-17, latency-associated peptide (LAP), perforin, granzyme B, CTLA-4, PD-1, CD39, and CD73. The analytical strategy is shown in **Supplementary Figures 1–5**.

### 2.9 Statistical analysis

The Shapiro–Wilk normality test was performed for all parameters evaluated using flow cytometry. If normally distributed, multiple comparisons were performed using two-way ANOVA and Tukey’s *post-hoc* test or one-way ANOVA and Tukey’s *post-hoc* test. Kruskal-Wallis with Dunn’s multiple comparisons test was performed if the data were not normally distributed. The immunological data between volunteers who had and had not developed certain symptoms in the Mild Recovered group was compared and analyzed based on the following clinical data: dyspnea, sore throat, nasal obstruction, myalgia, arthralgia, fatigue, diarrhea, anosmia, and ageusia. These comparisons were performed using one-way ANOVA and Tukey’s *post hoc* test or Kruskal-Wallis with Dunn’s multiple comparisons tests. Differences were considered statistically significant at p < 0.05. Statistical analyses were performed using GraphPad Prism 8.4.3. To create a heat map matrix regarding immunological marker expression in the HC, mild recovered, and severe recovered groups, we used Morpheus software and adjusted it to Z-score (22).

## 3 Results

### 3.1 Characteristics of HC, mild recovered, and severe recovered volunteers

For this study, we recruited 24 volunteers and distributed them into three groups. In the HC group, 8 volunteers (4 male and 4 female) with a mean age of 35.62 ( ± 8.63) were enrolled. The Mild Recovered group included 9 volunteers (5 male and 4 female) with a mean age of 33.22 ( ± 6.70). Finally, 7 volunteers were enrolled in the Severe Recovered group (4 male and 3 female) with a mean age of 36.85 ( ± 7.64) (**Tables 2**–**4**). No significant differences in age were found between the groups.

**Table 2:** Gender, age, and ethnic-racial self-classification of healthy control volunteers (HC).

#Code	Gender	Age	Ethnic-racial self-classification
**CTL 1**	Female	46	White
**CLT 3**	Male	47	White
**CLT 4**	Female	28	White
**CLT 5**	Male	35	White
**CLT 6**	Male	22	Brown
**CLT 7**	Female	36	Indigenous
**CLT 8**	Male	40	White
**CLT 9**	Female	31	Brown
**MEAN**	*4F:4M	**35.62 ( ± 8.63)**	

*F, Female; M, Male. The bold value represents the mean and standard deviation (in brackets) of the Age regarding HC volunteers.

**Table 3:** Gender, age, ethnic-racial self-classification, comorbidities, and symptoms of mild recovered volunteers (Mild Recovered).

#Code	Gender	Age	Blood collection after recovery of COVID-19 (days)	Ethnic-racial self-classification	Comorbidities	Symptoms
**COV3**	Male	21	194	Brown	Guillain-Barré	Headache, Runny nose, cough with phlegm, Nasal obstruction, Chills, and Fatigue
**COV5**	Female	33	204	Brown	Obesity	Fever, Headache, Dry cough, Myalgia, Chills, Fatigue, and Anosmia
**COV6**	Male	24	214	Brown		Fever, Headache, Runny nose, Sore throat, Dyspnea, Diarrhea, Fatigue, Anosmia, and Ageusia
**COV7**	Male	39	211	Brown		Fever and Dry cough
**COV8**	Male	32	195	Brown	Obesity	Runny nose, Sore throat, Nasal obstruction, Myalgia, Diarrhea, and Fatigue
**COV9**	Male	40	56	Brown		Headache, Runny nose, and Dyspnea
**COV10**	Female	36	16	White	Obesity	Arthralgia
**COV11**	Female	39	196	Brown		Fever, Headache, Runny nose, Sore throat, Dry cough, Dyspnea, Tachypnea, Arthralgia, Myalgia, Nausea, Vomiting, Diarrhea, Chills, Fatigue, Anosmia, and Ageusia
**COV15**	Female	35	50	Brown	Obesity	Headache, Runny nose, Sore throat, Dry cough, Cough with phlegm, Nasal obstruction, and Fatigue
**MEAN**	*4F:5M	**33.22 ( ± 6.70)**	**148.44 ( ± 81.83)**			

*F, Female; M, Male. The bold value represents the mean and standard deviation (in brackets) of the Age and Blood collection after recovery of COVID-19 (days) regarding mild recovered volunteers.

Most volunteers who experienced mild COVID-19 reported headache, runny nose, and fatigue (**Table 3**). All volunteers in the severe recovery group experienced pulmonary infiltration, low oxygen saturation, and headache, whereas almost all had high fever, tachypnea, and fatigue (**Table 4**). Regarding comorbidities, obesity was a significant chronic disease observed among 56% of the recovered COVID-19 cases.

**Table 4:** Gender, age, ethnic-racial self-classification, comorbidities, and symptoms of severe recovered volunteers (Severe Recovered).

#Code	Gender	Age	Blood collection after recovery of COVID-19 (days)	Ethnic-racial self-classification	Comorbidities	Symptoms
**COV1**	Female	27	73	Brown		Low blood oxygen saturation, Headache, Lung infiltrate, Sore throat, Dry cough, Dyspnea, Nasal obstruction, Tachypnea, Diarrhea, and Fatigue.
**COV2**	Male	43	182	Brown	Hypertension and Obesity	Fever, Low blood oxygen saturation, Headache, Arthralgia, Myalgia, Lung infiltrate, Nausea, Diarrhea, Fatigue, Tachypnea, Eye swelling, Anosmia, and Ageusia
**COV4**	Female	38	138	White	Hypertension and Obesity	Fever, Low blood oxygen saturation, Headache, Dry cough, Cough with phlegm, Dyspnea, Tachypnea, Myalgia, Vomiting, Lung infiltrate, Chills, and Fatigue
**COV14**	Male	25	301	Black	Obesity	Low blood oxygen saturation, Fever, Headache, Lung infiltrates, Runny nose, Sore throat, Dry cough, Dyspnea, Nausea, Fatigue, Eye swelling, and Anosmia
**COV16**	Male	43	40	Brown	Asthma and Obesity	Low blood oxygen saturation, Fever, Headache, Sore throat, Cough with blood, Lung infiltrates, dyspnea, and Tachypnea
**COV17**	Female	40	62	Brown		Fever, Headache, Sore throat, Low blood oxygen saturation, Arthralgia, Myalgia, Lung infiltrate, Chills, Fatigue, Anosmia, Ageusia, and Tachypnea
**COV18**	Male	42	61	White	Obesity (IMC 38.5)	Low blood oxygen saturation, Fever, Headache, Runny nose, Sore throat, Lung infiltrates, Dry cough, Dyspnea, Nasal obstruction, Tachypnea, Myalgia, Diarrhea, Chills, Fatigue, Anosmia, and Ageusia.
**MEAN**	*3F:4M	**36.85 ( ± 7.64)**	**122.42 ( ± 93.40)**			

*F, Female; M, Male. The bold value represents the mean and standard deviation (in brackets) of the Age and Blood collection after recovery of COVID-19 (days) regarding severe recovered volunteers.

Finally, volunteers were asked to self-identify themselves among the following ethnic–racial classifications: white, brown, black, yellow (Asian), and indigenous (Native American). Volunteers who self-classified themselves as brown and white comprised 58.33% and 33.33%, respectively, whereas those of black and indigenous self-classification comprised 4.1% each. Thus, the demographic of the HC group was white (62.5%), brown (25%), and indigenous (12.5%); that of Mild Recovered group was brown (88.88%) and white (11.11%), whereas that of Severe Recovered group was brown (57.14%), white (28.57%), and black (14.28%). The demographic and clinical details of the volunteers are shown in **Tables 2**–**4**.

### 3.2 Mild-recovered volunteers have elevated levels of Tregs

**Figure 1:**
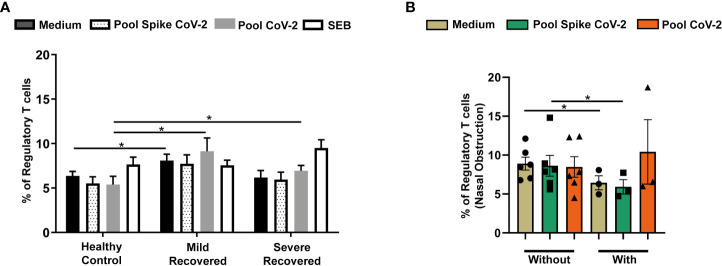
Frequency of regulatory T cells in peripheral blood from volunteers. Peripheral blood mononuclear cells (PBMC) were collected from volunteers not previously affected with SARS-CoV-2 infection (Healthy Control - HC, n = 8) and volunteers who had recovered from mild (Mild Recovered, n = 9) and severe (Severe Recovered, n = 7) COVID-19. The samples were incubated for 20 hours under four conditions: unstimulated (medium), stimulated with SARS-CoV-2 peptides (Pool Spike CoV-2 and Pool CoV-2 peptide), and stimulated with SEB. Unstimulated and SEB-stimulated PBMC were used as a negative and positive control, respectively. **(A)** Frequency of regulatory T cells in HC, Mild Recovered, and Severe Recovered. **(B)** Comparison of the frequency of regulatory T cells among Mild Recovered volunteers who had and had not experienced nasal obstruction during acute COVID-19. The symbols ●, ■, and ▲ represent each sample from Mild Recovered volunteer that was unstimulated (medium condition only) or stimulated with Pool Spike CoV-2 peptides or Pool CoV-2 peptides, respectively. Multiple comparisons were performed using two-way ANOVA and Tukey’s *post hoc* test, one-way ANOVA and Tukey’s *post hoc* test, and Kruskal-Wallis with Dunn’s multiple comparisons test when appropriate. The bars represent the mean values, and the error bars show the standard error of the mean (SEM) for each group. The lines above the bars indicate statistically significant differences between the two groups. *p < 0.05. SEB, staphylococcal enterotoxin B.

Analysis of PBMC from volunteers recovered from COVID-19 revealed a higher frequency of occurrence of Tregs in Mild Recovered group than in HC for non-stimulated samples (**Figure 1A**). In addition, pool CoV-2-stimulated samples from Mild Recovered and Severe Recovered groups presented more Tregs than did HC (**Figure 1A**). We also compared the frequency of occurrence of the studied lymphocyte subset among the Mild Recovered volunteers between those who had and had not experienced the following symptoms during acute COVID-19: dyspnea, myalgia, sore throat, arthralgia, fatigue, nasal obstruction, diarrhea, anosmia, and ageusia. Our data showed that those who did not experience nasal obstruction had more Tregs among the non-stimulated and Pool Spike CoV-2 stimulated PBMC than in those with this symptom (**Figure 1B**). A comparison of the frequency of occurrence of Treg among the Mild Recovered volunteers between those who had and had not experienced the other symptoms was also analyzed, but no significant results were found (**Supplementary Figure 1**).

### 3.3 Expression of IL-10 and IL-17 by Tregs is higher in volunteers who recovered from mild COVID-19

**Figure 2:**
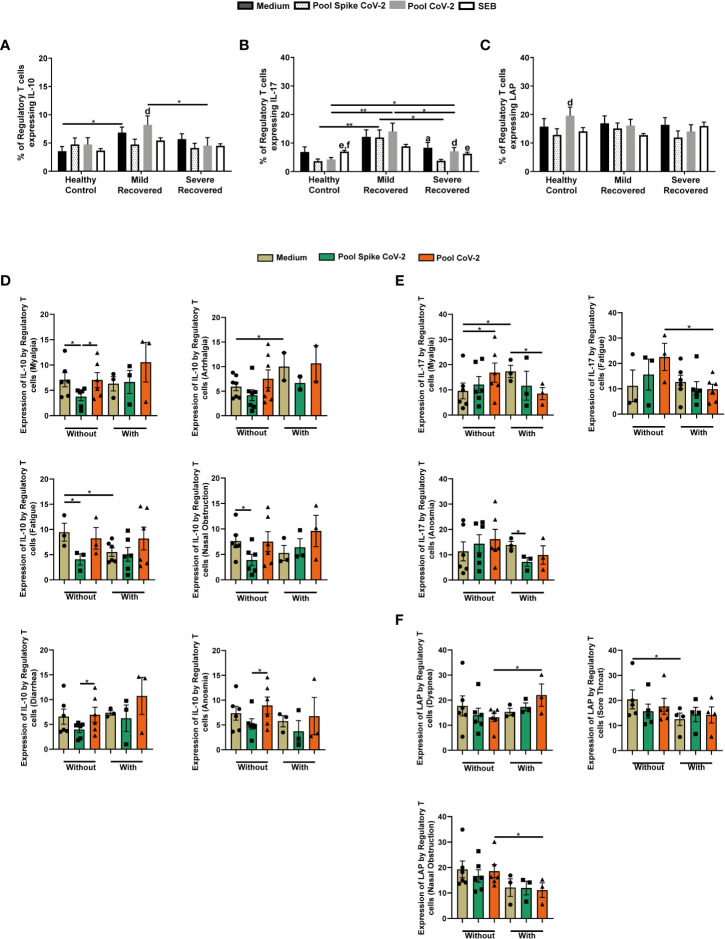
Regulatory T cells expressing IL-10, IL-17, and latency-associated peptide (LAP) in peripheral blood from volunteers. Peripheral blood mononuclear cells (PBMC) were collected from volunteers not previously affected by SARS-CoV-2 infection (Healthy Control – HC, n = 8) and volunteers who recovered from mild (Mild Recovered, n = 9) and severe (Severe Recovered, n = 7) COVID-19. The samples were incubated for 20 hours under four conditions: unstimulated (medium), stimulated with SARS-CoV-2 peptides (Pool Spike CoV-2 and Pool CoV-2 peptide), and stimulated with SEB. Unstimulated and SEB-stimulated PBMC were used as a negative and positive control, respectively. Frequency of regulatory T cells expressing IL-10 **(A)**, IL-17 **(B)**, and LAP **(C)** in HC, Mild Recovered, and Severe Recovered. Lowercase letters represent statistical differences (p < 0.05) between conditions within each group: (a) (medium × Pool Spike CoV-2), (d) (Pool Spike CoV-2 × Pool CoV-2), (e) (Pool Spike CoV-2 × SEB), and (f) (pool CoV-2 × SEB). **(D)** Comparison of the frequency of regulatory T cells expressing IL-10 among Mild Recovered volunteers who had and had not experienced the following symptoms: myalgia, arthralgia, fatigue, nasal obstruction, diarrhea, and anosmia during acute COVID-19. **(E)** Comparison of the frequency of regulatory T cells expressing IL-17 among Mild Recovered volunteers who had and had not experienced the following symptoms: myalgia, fatigue, and anosmia during acute COVID-19. **(F)** Comparison of the frequency of regulatory T cells expressing LAP among Mild Recovered volunteers who had and had not experienced the following symptoms: dyspnea, sore throat, and nasal obstruction during acute COVID-19. The symbols ●, ■, and ▲ represent each sample from Mild Recovered volunteer that was unstimulated (medium condition only) or stimulated with Pool Spike CoV-2 peptides or Pool CoV-2 peptides, respectively. Multiple comparisons were performed using two-way ANOVA and Tukey’s *post hoc* test, one-way ANOVA and Tukey’s *post hoc* test, and Kruskal-Wallis with Dunn’s multiple comparisons test when appropriate. The bars represent the mean values, and the error bars show the standard error of the mean (SEM) for each group. The lines above the bars indicate statistically significant differences between the two groups. *p < 0.05; **p < 0.01. SEB, staphylococcal enterotoxin B.

We also evaluated cytokine expression by Tregs in all the groups. Mild Recovered non-stimulated samples had higher levels of IL-10-producing Tregs than did the HC group (**Figure 2A**). Moreover, in Pool CoV-2-stimulated cells, the frequency of CD3^+^CD4^+^CD25^+^FOXP3^+^IL-10^+^ was lower in the Severe Recovered group than in the Mild Recovered group (**Figure 2A**). In the Mild Recovered group, the IL-10 expression by Tregs due to Pool CoV-2 peptide stimulus was higher compared with that due to Pool Spike CoV-2 stimulus (**Figure 2A**).

We also detected an elevated IL-17^+^ Treg frequency in PBMC from volunteers in the Mild Recovered group stimulated with SARS-CoV-2 related peptides compared with those from HC and Severe Recovered volunteers (**Figure 2B**). Severe Recovered group also presented higher IL-17 expression by Tregs than did the Pool CoV-2-stimulated PBMC in HC (**Figure 2B**). In addition, in HC samples incubated with SEB, IL-17^+^ Treg expression was more pronounced than in PBMC incubated with SARS-CoV-2-related peptides (**Figure 2B**). In contrast, Pool Spike CoV-2 reduced IL-17 expression by Tregs in the Severe Recovered group (**Figure 2B**). Finally, the levels of LAP^+^ Tregs did not differ between the HC, Mild Recovered, and Severe Recovered groups (**Figure 2C**). However, in the Pool CoV-2-stimulated PBMC, the frequency of Tregs expressing LAP was more accentuated than in Pool Spike CoV-2-stimulated samples (**Figure 2C**).

We also compared cytokine expression by Tregs among Mild Recovered volunteers between those who had and had not experienced certain symptoms of acute COVID-19. The Pool Spike CoV-2-stimulated samples of Mild Recovered volunteers who had not developed myalgia, fatigue, or nasal obstruction presented lower IL-10^+^ Treg frequency than did the unstimulated cells (**Figure 2D**). Diminished levels of IL-10 expression were observed in Pool Spike CoV-2-stimulated-Tregs compared with that of Pool CoV-2-stimulated Tregs in volunteers who had not developed myalgia, diarrhea, or anosmia during acute disease (**Figure 2D**). Finally, in unstimulated samples, the expression of IL-10 by Tregs was higher and lower in volunteers with arthralgia and fatigue, respectively, than in those who had not developed these symptoms (**Figure 2D**).

Interestingly, compared to unstimulated samples, Pool CoV-2 elicited the expression of IL-17 by Tregs in the Mild Recovered volunteers who did not present with myalgia and diminished the frequency of IL-17^+^ Treg in those who developed this symptom (**Figure 2E**). In the unstimulated samples, expression of IL-17 by Tregs from volunteers who did not have myalgia was lower than that by Tregs from those with this symptom (**Figure 2E**). Our data showed that IL-17 expression was less prevalent in volunteers with fatigue than in those without fatigue in samples stimulated with Pool CoV-2 (**Figure 2E**). For volunteers who experienced anosmia, the Pool Spike CoV-2 stimulus reduced the expression of IL-17 in Tregs compared with that of unstimulated samples (**Figure 2E**).

We also found elevated LAP^+^ Treg levels in Pool CoV-2-stimulated samples from Mild Recovered volunteers with dyspnea, whereas the opposite was observed in nasal obstruction (**Figure 2F**). In addition, unstimulated PBMC from Mild Recovered volunteers who had not experienced sore throat expressed a higher frequency of LAP-expressing Tregs than did the PBMC from those who had this symptom (**Figure 2F**). A comparison of cytokine expression among Mild Recovered volunteers between those who had and had not experienced the other symptoms was also analyzed, but no significant results were found (**Supplementary Figure 2**).

### 3.4 Production of cytotoxic granules by Tregs was higher in mild recovered volunteers who experienced dyspnea

**Figure 3:**
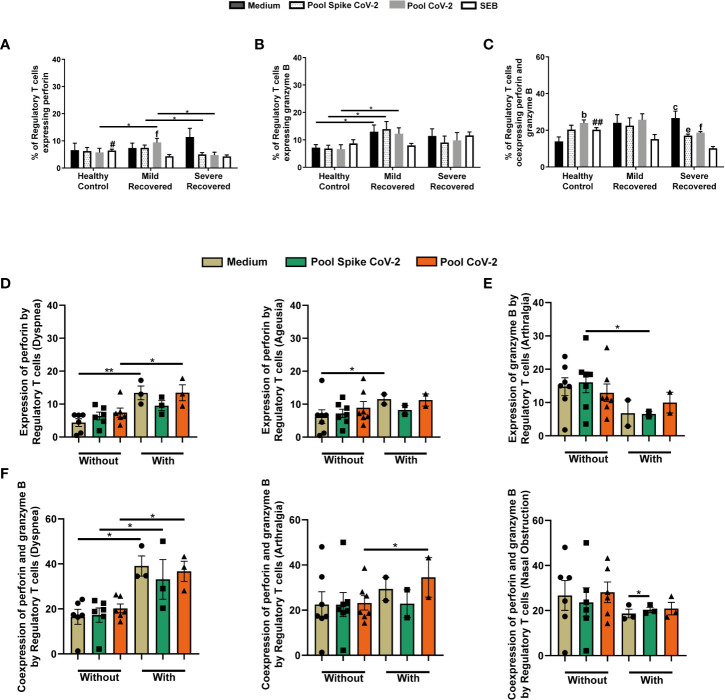
Regulatory T cells expressing perforin and granzyme B in peripheral blood from volunteers. Peripheral blood mononuclear cells (PBMC) were collected from volunteers not previously affected by SARS-CoV-2 infection (Healthy Control - HC, n = 8) and volunteers who recovered from mild (Mild Recovered, n = 9) and severe (Severe Recovered, n = 7) COVID-19. The samples were incubated for 20 hours under four conditions: unstimulated (medium), stimulated with SARS-CoV-2 peptides (Pool Spike CoV-2 and Pool CoV-2 peptide), and stimulated with SEB. Unstimulated and SEB-stimulated PBMC were used as a negative and positive control, respectively. Frequency of regulatory T cells expressing perforin **(A)**, granzyme **(B)**, and co-expressing perforin and granzyme B **(C)** in HC, Mild Recovered, and Severe Recovered. Lowercase letters represent statistical differences (p < 0.05) between the conditions within each group: (b) (medium × Pool CoV-2), (c) (medium × SEB), (e) (Pool Spike CoV-2 × SEB), and (f) (Pool CoV-2 × SEB). Symbol **#** represents statistically significant differences between the HC and Severe Recovered groups in the SEB conditions. The bars with **#** above depict the higher expression of the analyzed marker compared with that in the other group. **(D)** Comparison of the frequency of regulatory T cells expressing perforin among Mild Recovered volunteers who had and had not experienced the following symptoms: dyspnea and ageusia during acute COVID-19. **(E)** Comparison of the frequency of regulatory T cells expressing granzyme B among Mild Recovered volunteers who had and had not experienced arthralgia during acute COVID-19. **(F)** Comparison of the frequency of regulatory T cells coexpressing perforin and granzyme B among Mild Recovered volunteers who had and had not experienced the following symptoms: dyspnea, arthralgia, and nasal obstruction during acute COVID-19. The symbols ●, ■, and ▲ represent each sample from Mild Recovered volunteer, that was unstimulated (medium condition only) or stimulated with Pool Spike CoV-2 peptides or Pool CoV-2 peptides, respectively. Multiple comparisons were performed using two-way ANOVA and Tukey’s *post hoc* test, one-way ANOVA and Tukey’s *post hoc* test, and Kruskal-Wallis with Dunn’s multiple comparisons test when appropriate. The bars represent the mean values, and the error bars show the standard error of the mean (SEM) for each group. The lines above the bars indicate statistically significant differences between the two groups. *p < 0.05; **p < 0.01; ^#^p < 0,05; ^##^p < 0.01. SEB, staphylococcal enterotoxin B.

The present study also analyzed the production of cytotoxic granules by Tregs. Pool CoV-2-stimulated samples presented a higher Perforin^+^ Treg frequency in the Mild Recovered group than in the other groups (**Figure 3A**). In contrast, in Pool Spike CoV-2 stimulus Tregs in the Mild Recovered group produced more perforin than that in the Severe Recovered group (**Figure 3A**). In addition, SEB-incubated PBMC from HC presented higher levels of Perforin^+^ Tregs than in cells from Severe Recovered group (**Figure 3A**). Finally, in the Mild Recovered group, the frequency of Tregs that produced perforin was lower in the SEB-stimulated cells than in the Pool CoV-2-stimulated samples (**Figure 3A**).

We also found elevated levels of granzyme B producing Treg in Mild Recovered group than in the HC in unstimulated or SARS-CoV-2-related peptide-stimulated samples (**Figure 3B**). When perforin and granzyme B co-expression was analyzed, we observed that SEB-stimulated PBMC from HC presented higher levels of Tregs co-expressing both granules than did the cells from the Severe Recovered group (**Figure 3C**). In the HC, Pool CoV-2 stimulus induced a higher frequency of perforin^+^granzyme B^+^ Tregs compared to that in the unstimulated samples (**Figure 3C**). In contrast, in the Severe Recovered group, the SEB-incubated samples presented lower perforin and granzyme B co-expression by Tregs than in the other conditions (**Figure 3C**).

We compared and analyzed the expression of perforin by Tregs among Mild Recovered volunteers between those who had and had not experienced certain symptoms and found higher Perforin^+^ Treg levels in unstimulated and Pool CoV-2-stimulated PBMC from volunteers who presented with dyspnea during acute COVID-19 than in those who did not (**Figure 3D**). We also detected higher levels of perforin produced by unstimulated Tregs from volunteers who had ageusia than in those who did not (**Figure 3D**). Elevated granzyme B expression was observed in Tregs in samples stimulated with Pool Spike CoV-2 in Mild Recovered volunteers who did not have arthralgia during acute COVID-19 than in those who did (**Figure 3E**).

In samples unstimulated and stimulated with peptides related to SARS CoV-2, the co-expression of cytotoxic granules by Tregs was higher in Mild Recovered volunteers who experienced dyspnea than in those who did not (**Figure 3F**). Higher levels of Tregs Perforin^+^Granzyme B^+^ cells were detected in Pool CoV-2- stimulated PBMC from volunteers who had arthralgia than in those who did not (**Figure 3F**). However, for those who developed nasal obstruction during acute COVID-19, we observed a mild Pool Spike CoV-2-induced co-expression of cytotoxic granules by Tregs (**Figure 3F**). Comparison of the cytotoxic granule expression among Mild Recovered volunteers between those who had and had not experienced other symptoms were also analyzed, but no significant results were found (**Supplementary Figure 3**).

### 3.5 Pool spike CoV-2 stimulus reduces CTLA-4 expression by Tregs in mild recovered volunteers who did not experience certain symptoms

This study evaluated the expression of inhibitory receptors such as PD-1 and CTLA-4. Although CTLA-4 did not show significant changes in any of the studied groups (**Supplementary Table 1**), the frequency of Tregs expressing PD-1 was lower in the Severe Recovered group than in the Mild Recovered group in PBMC stimulated with Pool Spike CoV-2 (**Supplementary Table 1**). The Pool Spike CoV-2-stimulated samples in the Mild Recovered group presented a higher frequency of PD-1-producing Tregs than did those incubated with Pool CoV-2 peptide (**Supplementary Table 1**).

**Figure 4:**
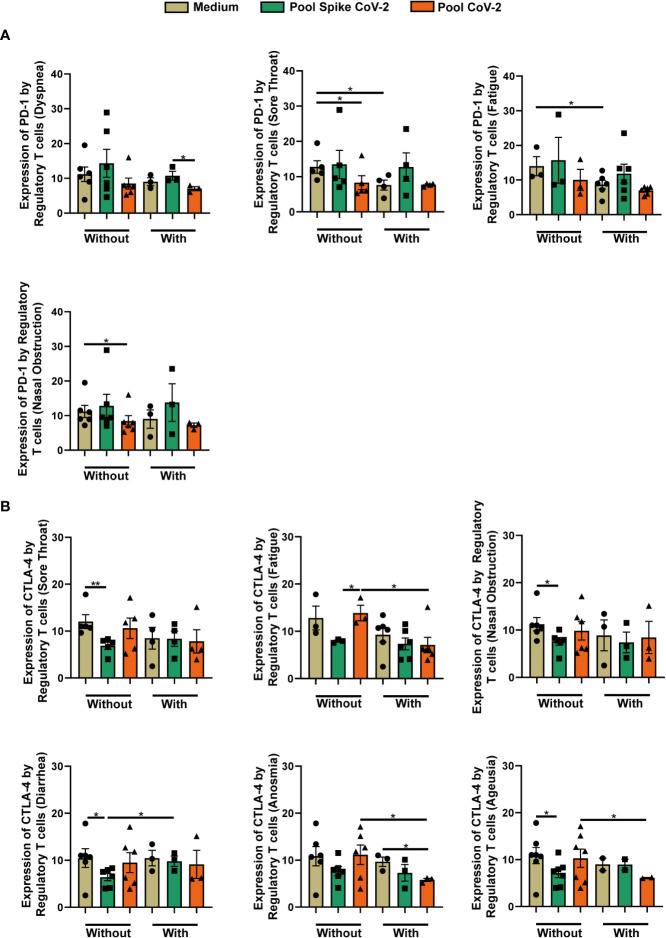
Comparison of the frequency of regulatory T cells expressing PD-1 and CTLA-4 among Mild Recovered volunteers who had and had not developed some symptoms. Peripheral blood mononuclear cells (PBMC) were collected from volunteers and the samples were incubated for 20 hours under four conditions: unstimulated (medium), stimulated with SARS-CoV-2 peptides (Pool Spike CoV-2 and Pool CoV-2 peptide), and stimulated with SEB (did not show in this figure). **(A)** Comparison of the frequency of regulatory T cells expressing PD-1 among Mild Recovered volunteers who had and had not developed the following symptoms: dyspnea, sore throat, fatigue, and nasal obstruction during acute COVID-19. **(B)** Comparison of the frequency of regulatory T cells expressing CTLA-4 among Mild Recovered volunteers who had and had not experienced the following symptoms: sore throat, fatigue, nasal obstruction, diarrhea, anosmia, and ageusia during acute COVID-19. The symbols ●, ■, and ▲ represent each sample from Mild Recovered volunteer that was unstimulated (medium condition only) or stimulated with Pool Spike CoV-2 peptides or Pool CoV-2 peptides, respectively. Multiple comparisons were performed using one-way ANOVA and Tukey’s *post hoc* test, and Kruskal-Wallis with Dunn’s multiple comparisons test when appropriate. The bars represent the mean values, and the error bars show the standard error of the mean (SEM) for each group. The lines above the bars indicate statistically significant differences between the two groups. *p < 0.05; **p < 0.01. SEB, staphylococcal enterotoxin B.

Analyzing the PD-1^+^ Treg frequency between Mild Recovered volunteers with or without certain symptoms revealed that those who experienced dyspnea presented lower levels of PD-1^+^ Tregs in PBMC stimulated with Pool CoV-2 than with Pool Spike CoV-2 (**Figure 4A**). In addition, the volunteers who did not develop sore throat or nasal obstruction presented a higher frequency of PD-1^+^ Treg in unstimulated PBMC than in those stimulated with Pool CoV-2 (**Figure 4A**). Finally, unstimulated samples from volunteers who did not experience sore throat or fatigue presented a higher frequency of PD-1 producing Tregs than the cells from volunteers who developed these symptoms (**Figure 4A**).

The frequency of CTLA-4^+^ Treg among Mild Recovered volunteers between those who had and had not experienced certain symptoms during acute COVID-19 was compared, and we observed that those who had not experienced sore throat, nasal obstruction, diarrhea, and ageusia had a lower Pool Spike CoV-2 peptide-induced CTLA-4 expression in Tregs compared with that in unstimulated cells (**Figure 4B**). We observed higher levels of CTLA-4^+^ Tregs in PBMC stimulated with Pool CoV-2 than with Pool Spike CoV-2 among Mild Recovered volunteers who had not experienced fatigue (**Figure 4B**). Pool CoV-2-stimulated PBMC presented fewer Tregs expressing CTLA-4 in those who had developed anosmia when compared with that of the unstimulated samples (**Figure 4B**). We also observed a lower Treg CTLA-4^+^ frequency in Pool CoV-2-stimulated PBMC from Mild Recovered volunteers who had experienced fatigue, anosmia, and ageusia than in those who had not developed these symptoms (**Figure 4B**). The Treg CTLA-4^+^ levels in the Pool Spike CoV-2-stimulated PBMC were higher in volunteers who had developed diarrhea than in those who had not (**Figure 4B**). A comparison of the expression of the inhibitory receptors among Mild Recovered volunteers between those who had and had not experienced other symptoms was also analyzed, but no significant results were found (**Supplementary Figure 4**).

### 3.6 Contrary CD39 and CD73 expression patterns are observed among mild recovered volunteers between those who had and had not experienced certain symptoms

**Figure 5:**
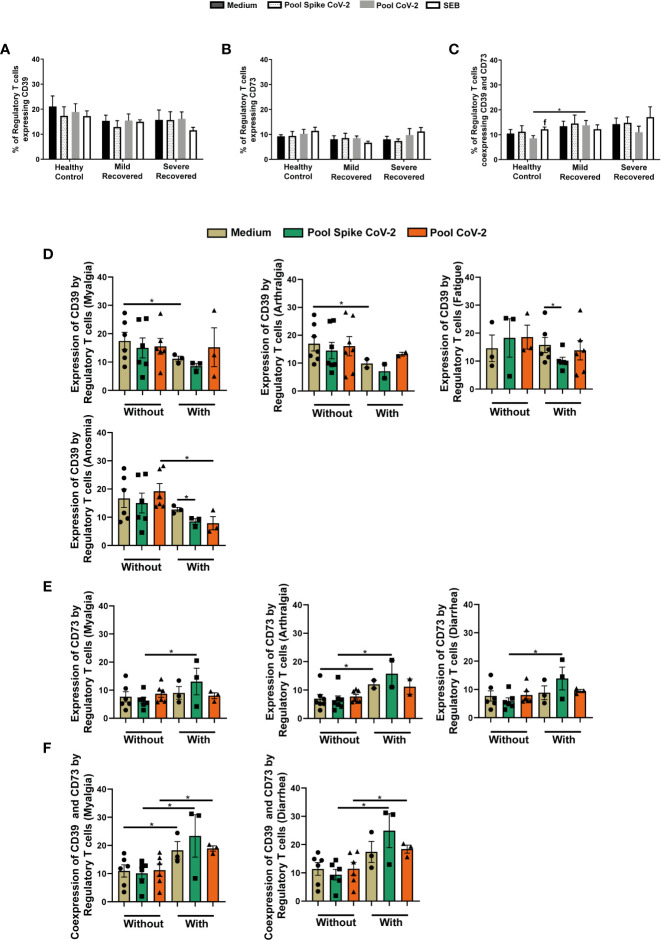
Regulatory T cells expressing CD39 and CD73 in peripheral blood from volunteers. Peripheral blood mononuclear cells (PBMC) were collected from volunteers not previously affected by SARS-CoV-2 infection (Healthy Control - HC, n = 8) and volunteers who recovered from mild (Mild Recovered, n = 9) and severe (Severe Recovered, n = 7) COVID-19. The samples were incubated for 20 hours under four conditions: unstimulated (medium), stimulated with SARS-CoV-2 peptides (Pool Spike CoV-2 and Pool CoV-2 peptide), and stimulated with SEB. Unstimulated and SEB-stimulated PBMC were used as a negative and positive control, respectively. Frequency of regulatory T cells expressing CD39 **(A)**, CD73 **(B)**, and co-expressing CD39 and CD73 **(C)** in HC, Mild Recovered, and Severe Recovered. Lowercase letters represent statistical differences (p < 0.05) between the conditions within each group: (f) (Pool CoV-2 × SEB). **(D)** Comparison of the frequency of regulatory T cells expressing CD39 among Mild Recovered volunteers who had and had not experienced the following symptoms: myalgia, arthralgia, fatigue, and anosmia during acute COVID-19. **(E)** Comparison of the frequency of regulatory T cells expressing CD73 among Mild Recovered volunteers who had and had not experienced the following symptoms: myalgia, arthralgia, and diarrhea during acute COVID-19. **(F)** Comparison of the frequency of regulatory T cells coexpressing CD39 and CD73 among Mild Recovered volunteers who had and had not experienced the following symptoms: myalgia and diarrhea during acute COVID-19. The symbols ●, ■, and ▲ represent each sample from Mild Recovered volunteer that was unstimulated (medium condition only) or stimulated with Pool Spike CoV-2 peptides or Pool CoV-2 peptides, respectively. Multiple comparisons were performed using two-way ANOVA and Tukey’s *post hoc* test, one-way ANOVA and Tukey’s *post hoc* test, and Kruskal-Wallis with Dunn’s multiple comparisons test when appropriate. The bars represent the mean values, and the error bars show the standard error of the mean (SEM) for each group. The lines above the bars indicate statistically significant differences between the two groups. *p < 0.05. SEB, staphylococcal enterotoxin B.

Finally, the expression of CD39 and CD73 by Tregs was analyzed. As shown in **Figures 5A, B**), no significant changes in the levels of Tregs expressing CD39 or CD73 were observed between the studied groups. However, when the co-expression of CD39^+^CD73^+^ was analyzed, Mild Recovered samples incubated with Pool CoV-2 had a higher frequency of CD39^+^CD73^+^ Treg expression than did the HC (**Figure 5C**). In the HC group, we also observed the high co-expression by Tregs in samples stimulated with SEB that with Pool CoV-2 (**Figure 5C**).

The comparison of ectonucleotidase expression among Mild Recovered volunteers between those who had and had not experienced certain symptoms showed a higher CD39^+^ Treg frequency in unstimulated PBMC from volunteers who had not developed myalgia and arthralgia than in those who had (**Figure 5D**). In addition, the levels of CD39 expression by Tregs in volunteers who had presented with fatigue and anosmia were more prevalent in the unstimulated samples than in Pool Spike CoV-2-stimulated samples (**Figure 5D**). The anosmia-positive volunteers were also marked by a lower expression of CD39 by Tregs in Pool CoV-2 stimulus compared to those that had not experienced this symptom (**Figure 5D**).

In Pool Spike CoV-2-incubated samples from Mild Recovered volunteers who had myalgia, arthralgia, and diarrhea, we observed a higher level of CD73^+^ Treg frequency than in those who had not presented these symptoms (**Figure 5E**). Volunteers who experienced arthralgia also presented higher CD73 expression by Tregs in unstimulated samples than in those without arthralgia (**Figure 5E**). Interestingly, CD39^+^CD73^+^ Tregs were more prevalent in all conditions for volunteers who had developed myalgia than for those who had not (**Figure 5F**). A similar result was observed for diarrhea with the exception of unstimulated samples; no significant differences were found among volunteers between those who had and had not developed this symptom (**Figure 5F**). No significant results were found in the comparison of ectonucleotidase expression among Mild Recovered volunteers between those who had and had not experienced other symptoms (**Supplementary Figure 5**).

## 4 Discussion

The worldwide emergency caused by the current COVID-19 pandemic has led to significant efforts by the scientific community to understand the host immune response to SARS-CoV-2 infection (5). Although most infected individuals develop mild COVID-19, the severity was high in some people, and excessive inflammation is usually associated with the worsening of the illness (23, 24). To better understand the impact of SARS-CoV-2 infection on adaptive immunity after infection resolution, we evaluated the peripheral Tregs of volunteers who had recovered from mild and severe COVID-19. Compared to HC, we report higher levels of Tregs in Pool CoV-2-stimulated samples from Mild and Severe Recovered volunteers. In addition, unstimulated PBMC from the Mild Recovered group presented a higher frequency of Tregs than did HC, suggesting long-term immune changes after SARS-CoV-2 infection as previously reported by Liu et al. (2021) (25). The absence of statistical differences between the Mild and Severe Recovered groups could indicate no correlation between Treg frequency and the course of COVID-19.

However, it is interesting to consider the possibility that after recovery from COVID-19, the Treg levels were downregulated. This raises questions regarding the parameters and their actual impact on distinct forms of the disease. However, the higher Treg frequency in unstimulated PBMC from the Mild Recovered group than in HC suggests improved activity during the acute stage that was partially sustained after the cure. In this case, no differences were found between the Mild Recovery volunteers who did and did not have the evaluated symptoms during acute disease, nasal obstruction being an exception. Volunteers without nasal obstruction had more Tregs than in those with this clinical sign, indicating that these cells participate in the development of an attenuated form of COVID-19. However, the analysis of cell frequency alone does not provide details on the impact of long-term SARS-CoV-2 infection on Tregs from recovered volunteers. Thus, we analyzed the expression of some proteins related to Treg activity in all the evaluated groups.

IL-10 is a cytokine with anti-inflammatory properties used by Tregs as a direct tool for immune response modulation during immunosuppressive activity (26). We found a higher frequency of IL-10^+^ Treg in unstimulated or Pool CoV-2-stimulated Mild Recovered PBMC compared to that in HC and Severe Recovered groups, strengthening the hypothesis of persistent immune changes after the resolution of infection (27, 28). Although some studies correlate cytokines such as IL-6 and IL-10 as predictors of COVID-19 severity, elevated IL-10 levels could act to moderate excessive inflammation even though it is not able to do so (29–31).

Considering that acute COVID-19 induced higher expression levels of Treg IL-10^+^ in Mild Recovered volunteers, these results may indicate an improved regulatory activity to aid in an adequate antiviral response leading to the resolution of infection with little tissue injury. Consistent with this, Shuwa et al. (2021) (27) showed that GcPC-stimulated PBMC from convalescent patients with chest X-ray abnormalities manifested a reduced frequency of IL-10-producing B cells compared with that in those without the abnormalities, suggesting a correlation between pulmonary pathology resolution and B IL-10^+^ cell expansion. Volunteers who recovered from mild COVID-19 presented elevated levels of IL-10 producing Tregs, which points to a sustained immunosuppressive microenvironment that can attenuate inflammation upon reinfection. Our data showed that Pool CoV-2 stimulation elicited more IL-10 expression than did Pool Spike CoV-2 stimulus in the Mild Recovered group. This result points to increased suppressive capacity in reactive Tregs because Mild Recovered IL-10^+^ Tregs levels were also higher than that in the cells from Pool CoV-2 stimulated Severe Recovered group. Meckiff et al. (2020) (32) suggested a potentially impaired Treg response in hospitalized COVID-19 patients, corroborating our findings.

Analyzing the expression of IL-17 by Tregs (33–37), we observed a more pronounced level of cytokine synthesis in Mild Recovered volunteers. Some studies point to the contribution of this interleukin into the severity of COVID-19, also showing that the activation of IL-17 signaling pathways due to the interaction of SARS-CoV-2 ORF8 with IL-17 receptor could induce the expression of pro-inflammatory factor, contributing to cytokine storm (38, 39). However, Ghazavi et al. (2021) (40) found higher IL-17 plasma levels in patients with mild COVID-19, whereas Shuwa et al. (2021) (27) observed the expression of IL-17 among CD4+ T cells in a population of stimulated PBMC during acute disease, and it was maintained in the convalescent stage regardless of the clinical profile. Hence, it might be possible that SARS-CoV-2 infection elicited a greater IL-17 expression in Tregs from Mild Recovered volunteers during the acute stage and the levels were maintained after disease resolution. This could be why statistically significant differences were detected only after stimulation by SARS-CoV-2-related peptides. The similarity in IL-17 production between unstimulated, Pool Spike CoV-2-stimulated, and Pool CoV-2-stimulated cells supports the above-mentioned hypothesis.

The elevated levels of IL-17 synthesis by Tregs in Mild Recovered volunteers could also indicate a high level of expression of transcription factor (TF) RORγt. RORγt^+^ Tregs could possibly impede Th17 differentiation, thus attenuating the production of inflammatory mediators. Previous studies have suggested that Tregs expressing TF of other CD4^+^ T subpopulations help in the immunosuppression of distinct inflammatory profiles (36, 41–44). However, SARS-CoV-2 peptides appear to elicit a higher Th1 response profile in T cells (45–47). Hence, the decreased IL-17 expression by Tregs under Spike stimulus may point to an appropriate assembly of the antiviral immune response during reinfection in Severe Recovered volunteers, contributing to a faster infection resolution. However, both hypotheses suggest a distinct Treg profile between Mild and Severe Recovered volunteers with the potential to fight SARS-CoV-2 reinfection appropriately.

The contribution of IL-17 to proper disease resolution upon first exposure to or reinfection with SARS-CoV-2 could aid in understanding other potential favorable properties of IL-17^+^ Tregs. A study by Schnoeller et al. (2014) (48) demonstrated a protective role of IL-17 in respiratory syncytial virus (RSV) infection following vaccination with attenuated *Bordetella pertussis.* Wang et al. (2011) (49) also reported a beneficial role of IL-17 against the H5N1 virus, possibly through migratory modulation of B lymphocytes. Under certain conditions, Treg subpopulations express IL-17 displaying considerable and expressive immunosuppressive activity (35, 50, 51). Interestingly, *in vitro* studies by Li et al. (2012) (51) demonstrated that IL-17-producing Tregs, named Tr17, expressed higher amounts of perforin, granzyme B, and CD107a, suggesting a greater degranulation capacity compared with that of IL-17^-^Tregs. Taken together, our data indicate that the Treg subpopulation may improve the immunomodulatory profile in volunteers who recovered from mild COVID-19.

Consistent with Li et al. (2012) (51), IL-17^+^ Tregs may have been partially responsible for the higher expression of perforin and granzyme B found in the Mild Recovered group and probably contributed to the mildness of the disease in these volunteers during acute COVID-19. Interestingly, comparative analysis of data in the Mild Recovered group showed higher perforin levels or co-production of perforin and granzyme B by Tregs from those who experienced certain symptoms, especially dyspnea. Although cytotoxic granule expression may correlate with COVID-19 severity, perforin and granzyme production by Tregs is essential for its immunosuppressive properties (52–55). Considering the immunomodulatory roles of Tregs, the differential expression of cytotoxic granules in Mild Recovered volunteers affected mainly by dyspnea suggests a partial contribution of Tregs to inflammation control through this mechanism, resulting in milder forms of COVID-19. Loebbermann et al. (2012) (53) demonstrated the essential role of granzyme B-producing Treg cells in controlling pulmonary inflammation during acute viral infection. Hence, cytotoxic granule producing Tregs could exert an immunomodulatory effect through targeted immune cell death and prevent exacerbated inflammatory activity. The importance attributed to the Treg-secreted granules in inducing cell death (or suppression) in dendritic cells (DC), NK cells, and CD8+ T cells, raises the possibility of immunomodulation at the level of innate and adaptive immunity (52, 56, 57).

Interestingly, our data also showed a significant increase in Tregs expressing LAP and co-expressing perforin and granzyme B in Pool CoV-2-stimulated PBMC from HC. Samples of patients not previously exposed to the virus showed the presence of reactive T cells to SARS-CoV-2, probably due to exposure to other endemic coronaviruses (58). Therefore, our findings may point to immunological cross-reactivity caused by prior infections by other pathogens.

Modulation of costimulatory (such as CD28 and ICOS) or inhibitory (such as CTLA-4 and PD-1) receptors is a valuable mechanism for T lymphocyte responsiveness control (59, 60). Tregs express inhibitory receptors responsible for the modulation of the immune response through cell–cell contact mechanisms (55, 61). Despite similar CTLA-4 production levels among the evaluated groups, comparative analysis of the data of the Mild Recovered group demonstrated a decreased expression pattern of IL-10^+^ and CTLA-4^+^ Tregs in Pool Spike CoV-2-stimulated samples from volunteers who had not experienced specific symptoms. This result suggests a distinct modulation of immunosuppressive Treg tools among Mild Recovered volunteers who had and did not develop certain clinical signs during acute COVID-19. Some studies have indicated that SARS-CoV-2 peptides elicit a stronger Th1 response (45–47). Based on the differential Treg profile data, it seems possible that Mild Recovered volunteers experience a well-coordinated immune response, partially because of attenuated Treg immunosuppressive mechanisms, which elicited a faster antiviral signature. Considering the highly functional and balanced T cell responsiveness in asymptomatic individuals (62), our data suggest that immunomodulation at Treg levels aids in better infection resolution.

We also demonstrated that Pool Spike CoV-2 induced a higher frequency of PD-1^+^ Tregs than did Pool CoV-2 in Mild Recovered volunteers, similar to that observed in volunteers from the same group who had experienced dyspnea during acute COVID-19. Kamada et al. (2019) (63) reported an improvement in cell-mediated immunosuppression after blocking PD-1 on Tregs. Since the frequency of Tregs expressing PD-1 was more pronounced in Mild than in Severe Recovered samples with Pool Spike CoV-2 stimulation, these results indicate a possible limiting immunosuppressive activity that favors antiviral responsiveness.

Finally, we evaluated the expression of ectonucleotidases CD39 (NTPDase1) and CD73 (5′-ribonucleotide phosphohydrolase), which is another immunosuppressive mechanism exerted by Tregs through purinergic signaling modulation by hydrolysis of extracellular ATP (pro-inflammatory) and generation of adenosine (immunosuppressive) (55, 64–66). Even without significant differences in CD39 or CD73 expression among the recovered groups, the Pool CoV-2-stimulated PBMC from Mild Recovered volunteers presented more CD39^+^CD73^+^ Tregs than did the HC, suggesting greater adenosine synthesis capacity upon reinfection.

Interestingly, we found higher CD3^+^CD4^+^CD25^+^FOXP3^+^CD39^+^ T cell frequency in unstimulated conditions in Mild Recovered volunteers who had not experienced musculoskeletal symptoms (arthralgia and myalgia) during acute COVID-19. Past studies have shown an overall correlation between CD39 expression in some lymphocytes and excellent responsiveness to rheumatoid arthritis (RA)-associated treatments or between reduced RA activity and rheumatoid factor (RF) levels (67–69). In contrast, Gu et al. (2017) (70) showed that CD39^high^ Tregs may have more stability and protective roles compared with that CD39^low^ Tregs. This partially explains the higher frequency of CD39^+^ Tregs in Mild Recovered volunteers who had not experienced musculoskeletal pain during active disease. Based on these observations by Gu et al. (2017) (70), CD39^+^ Tregs could be evaluated to validate whether these cells are endowed with higher immunosuppressive capacity in volunteers without arthralgia and myalgia than in those who had developed these symptoms. This is a possibility considering that in Mild Recovered volunteers who had experienced anosmia and fatigue, Pool Spike CoV-2 elicited a downregulation in CD39^+^ Tregs. At the same time, a lower frequency of this subpopulation was found post Pool CoV-2 stimulation in samples of Mild Recovered volunteers who had experienced anosmia compared with those who had not.

The frequency of CD73^+^ Tregs, in contrast to that of CD39, was higher in Mild Recovered volunteers who had developed myalgia and arthralgia in the acute phase of the disease, suggesting differential ectonucleotidase modulation in the face of musculoskeletal symptoms. A correlation exists between low CD73 expression and the severity of juvenile idiopathic arthritis (JIA) (71). CD73 also plays a protective role in collagen-induced arthritis (CIA) in a mouse model for the study of RA (72). Thus, Mild Recovered COVID-19 volunteers who had experienced musculoskeletal symptoms possibly had better adenosine production mediated by Tregs. These results suggest a protective role of this subpopulation in this group, partially explaining the greater CD39^+^CD73^+^ Treg frequency in Mild Recovered volunteers who had developed myalgia.

Unfortunately, due to the lack of a longitudinal study, we could not determine whether the lower levels of CD39^+^ Tregs and higher levels of CD73^+^ and CD39^+^ CD73^+^ Tregs in volunteers who had musculoskeletal symptoms occurred before, during, or after the first infection. Since similar levels of CD39^+^ Tregs were observed in both stimulated and unstimulated PBMC, the frequency of this subpopulation in Mild Recovered volunteers without musculoskeletal symptoms could have been high even before the infection, leading to the absence of these clinical signs. This raises the possibility of a correlation between CD39 expression by Tregs and the course of COVID-19. We, however, suggest that Mild Recovered volunteers who had and had not experienced certain symptoms are endowed with distinct immunosuppressive components that can converge to aid in host protection in case of reinfection. The lower CD39^+^ and higher CD73^+^ Treg expression in Mild Recovered volunteers who had experienced certain symptoms suggests that the opposite expression of both enzymes possibly elicited by SARS-CoV-2 infection in the Mild Recovered group can persist for long periods after disease resolution.

## 5 Conclusion

In the present study, we observed higher frequencies of Tregs and expression of IL-10, IL-17, perforin, granzyme B, PD-1, and CD39^+^CD73^+^ co-expression by Tregs in the Mild Recovered group compared with that in HC or Severe Recovered group under certain conditions. We also observed a differential frequency of Tregs expressing immunological markers between cells from Mild Recovered volunteers among those who had and had not developed certain symptoms during acute COVID-19. Hence, we arrived at a few inferences. In Mild Recovered volunteers who had not experienced certain clinical signs, Pool Spike CoV-2 stimulus elicited a downregulation of CTLA-4^+^ and IL-10^+^ Tregs frequency, indicating attenuation of specific immunosuppressive mechanisms that possibly contributed to the effective antiviral response. A differential expression between CD39 and CD73 by Tregs was also observed. Dyspnea was associated with high levels of perforin and cytotoxic granule co-expression by the studied lymphocytes. Finally, the SARS-CoV-2-related peptides (Pool Spike CoV-2 and/or Pool CoV-2) induced changes in the expression of IL-10 and PD-1 in volunteers who recovered from mild COVID-19, IL-17 in volunteers who recovered from Severe COVID-19, and LAP and co-expression of cytotoxic granules in HC.

Our study has some limitations, including the small number of volunteers, mainly in the Severe Recovered group. This work does not encompass the full scale of heterogeneity in the Brazilian population. Hence, we were not able to explore genetics and age as factors that may affect the immune response to infections (73, 74). However, despite these limitations, our study reveals important data about the participation of Tregs in COVID-19, suggesting that modulation of immunosuppressive markers could partially drive a mild disease, and these alterations that possibly occur during the acute disease can persist for months. It is noteworthy to highlight that this study enrolled just unvaccinated volunteers. Thus, we believe that this study would greatly aid the scientific community to make further advancements and gain knowledge regarding the contribution of immunological changes at the level of Tregs to the development of distinct clinical profiles in COVID-19.

## Data availability statement

The original contributions presented in the study are included in the article/**Supplementary Material**. Further inquiries can be directed to the corresponding author.

## Ethics statement

The studies involving human participants were reviewed and approved by National Commission of Ethics in Research (certificate CAAE: 31354720.0.0000.5188). The patients/participants provided their written informed consent to participate in this study.

## Author contributions

PP: Investigation, data curation, formal analysis, visualization, and writing of the original draft. RP: Investigation and data curation. BC: Investigation and data curation. IM: Formal analysis and resources provided. FA: Formal analysis and resources provided. RV: Formal analysis. DJ: Formal analysis. IA: Writing, review, and editing. TK: Conceptualization, methodology, validation, resources provision, supervision, project administration, funding acquisition, writing of the original draft, review, and editing. All authors contributed to the article and approved the submitted version.
