# Supplementary material for: Differential regulatory T cell signature after recovery from mild COVID-19
Source: Front Immunol. 2023 Mar 8;14:1078922. doi: 10.3389/fimmu.2023.1078922 (PMC10030602; doi:10.3389/fimmu.2023.1078922)
Supplement: Supplementary file 1 [file Table_1.docx]

**Supplementary Table 1.** Regulatory T cells expressing PD-1 and CTLA-4 in peripheral blood from volunteers.

| **PD-1** | **Healthy Control** | **Mild Recovered** | **Severe Recovered** |
| --- | --- | --- | --- |
| **Medium** | 9.35 ± 4.79 | 10.45 ± 4.28 | 9.93 ± 4.46 |
| **Pool Spike CoV-2** | 9.03 ± 5.87 | 13.13 ± 8.00**^# d^** | 6.92 ± 4.48**^#^** |
| **Pool CoV-2** | 8.43 ± 4.48 | 8.02 ± 3.16 **^d^** | 10.08 ± 7.98 |
| **SEB** | 9.68 ± 4.02 | 9.73 ± 2.90 | 7.56 ± 1.42 |
| **CTLA-4** | **Healthy Control** | **Mild Recovered** | **Severe Recovered** |
| **Medium** | 9.44 ± 5.86 | 10.44 ± 4.10 | 10.90 ± 6.48 |
| **Pool Spike CoV-2** | 9.18 ± 5.70 | 7.53 ± 2.45 | 10.13 ± 6.42 |
| **Pool CoV-2** | 7.89 ± 3.56 | 9.36 ± 4.84 | 8.55 ± 6.48 |
| **SEB** | 9.74 ± 3.09 | 8.28 ± 1.83 | 10.22 ± 2.47 |

Table with a mean (±Standard Deviation) of PD-1 and CTLA-4 expression by regulatory T cells. Peripheral blood mononuclear cells (PBMC) were collected from volunteers not previously affected by SARS-CoV-2 infection (Healthy Control - HC, n = 8) and volunteers who recovered from mild (Mild Recovered, n = 9) and severe (Severe Recovered, n = 7) COVID-19. The samples were incubated for 20 hours under four conditions: unstimulated (medium), stimulated with SARS-CoV-2 peptides (Pool Spike CoV-2 and Pool CoV-2 peptide), and stimulated with SEB. Unstimulated and SEB-stimulated PBMC were used as a negative and positive control, respectively. Lowercase letters represent statistical differences (p < 0.05) between the conditions within each group: **d** (Pool Spike CoV-2 × Pool CoV-2). Symbol **#** represents statistically significant differences (p < 0.05) between the Mild Recovered and Severe Recovered groups in the Pool Spike CoV-2 conditions. Multiple comparisons were performed using two-way ANOVA and Tukey’s post hoc test, one-way ANOVA and Tukey’s post hoc test, and Kruskal-Wallis with Dunn’s multiple comparisons test when appropriate. SEB, staphylococcal enterotoxin B.
