# Supplementary material for: Differential regulatory T cell signature after recovery from mild COVID-19
Source: Front Immunol. 2023 Mar 8;14:1078922. doi: 10.3389/fimmu.2023.1078922 (PMC10030602; doi:10.3389/fimmu.2023.1078922)
Supplement: Supplementary file 2 [file DataSheet_1.pdf]

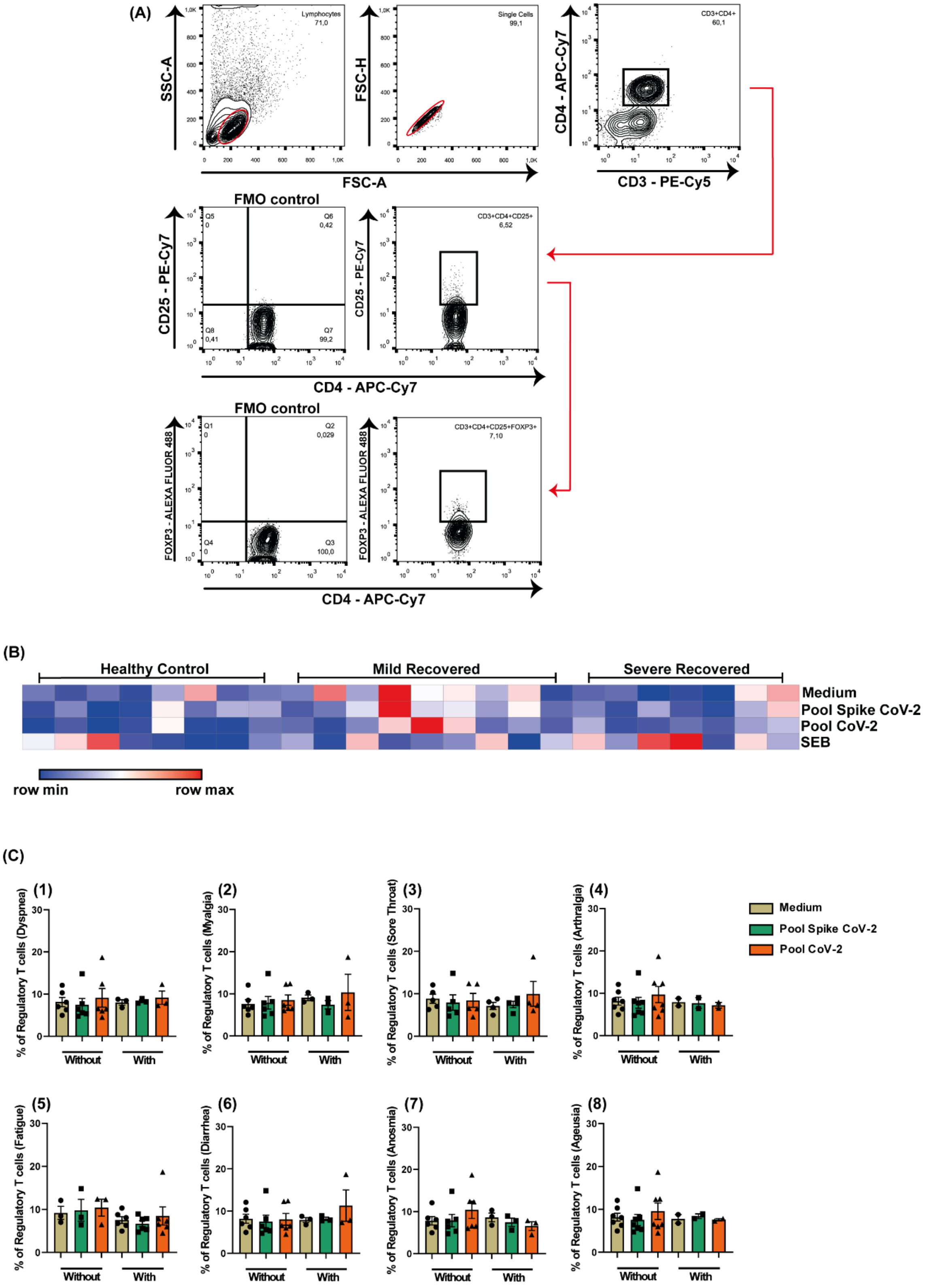

Supplementary Figure 1. Frequency of regulatory T cells in peripheral blood from volunteers. Peripheral blood mononuclear cells (PBMC) were collected from volunteers not previously affected

with SARS-CoV-2 infection (Healthy Control - HC, n = 8) and volunteers who had recovered from mild (Mild Recovered, n = 9) and severe (Severe Recovered, n = 7) COVID-19. **(A)** The strategy of analysis for regulatory T cell identification and counting. The representative dot plots were derived from unstimulated samples. **(B)** Heatmap of frequency of regulatory T cells in volunteers from the HC, Mild Recovered, and Severe Recovered groups based on flow cytometry analyses. Each row indicates the condition in which the PBMCs were submitted (shown on the right). Each column indicates a volunteer enrolled in the study. **(C.1-8)** Comparison of the frequency of regulatory T cells among Mild Recovered volunteers who had and had not experienced the following symptoms: dyspnea, myalgia, sore throat, arthralgia, fatigue, diarrhea, anosmia, and ageusia during acute COVID-19.

The symbols ●, ■, and ▲ represent each sample from Mild Recovered volunteer that was unstimulated (medium condition only) or stimulated with Pool Spike CoV-2 peptides or Pool CoV-2 peptides, respectively. When appropriate, multiple comparisons were performed using one-way ANOVA and Tukey’s post hoc test or Kruskal-Wallis with Dunn’s multiple comparisons tests. The bars represent mean values, and the error bars show the standard error of the mean (SEM) for each group.

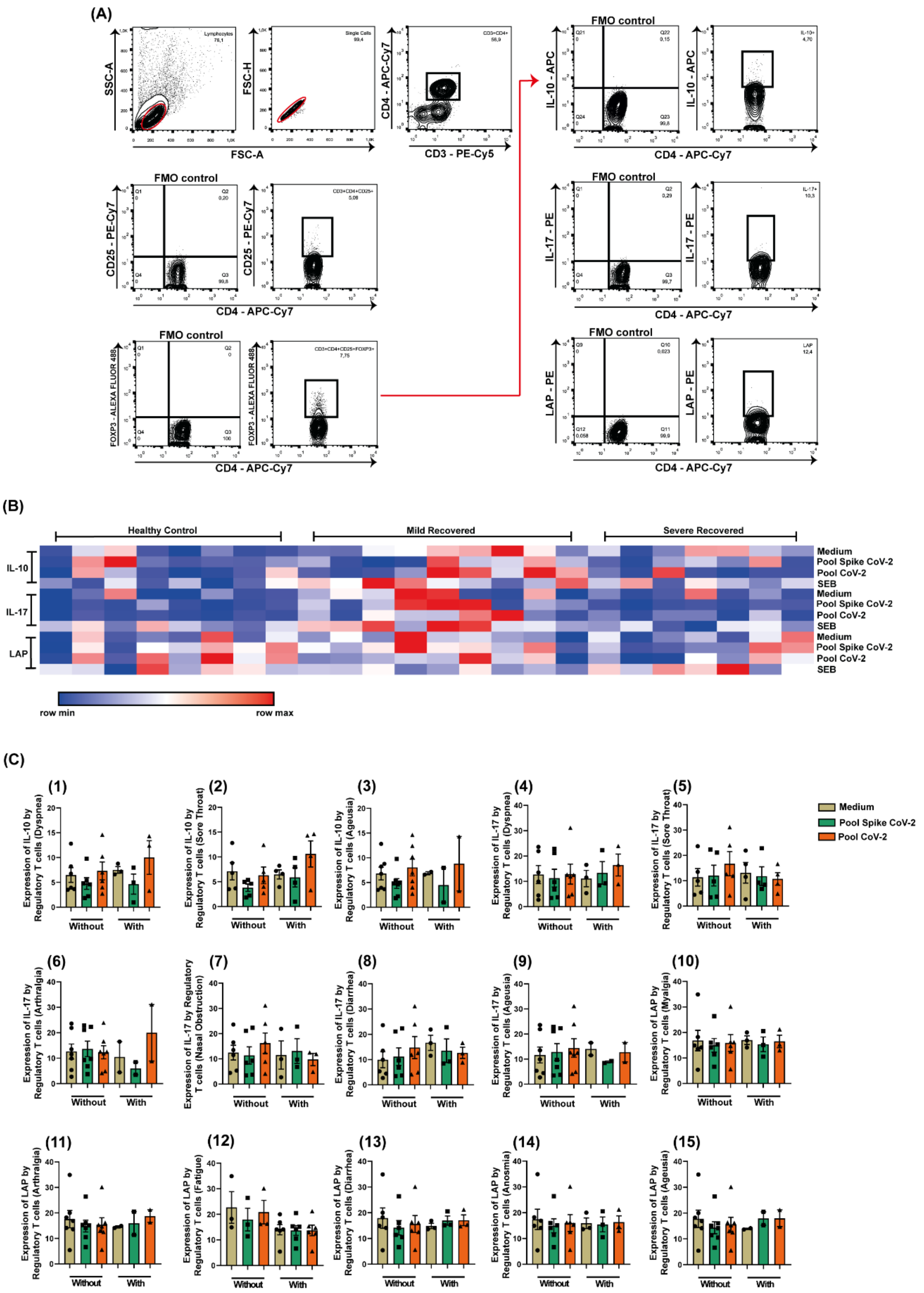

Supplementary Figure 2. **Regulatory T cells expressing IL-10, IL-17, and latency-associated peptide (LAP) in peripheral blood from volunteers**. Peripheral blood mononuclear cells (PBMC) were collected from volunteers not previously affected by SARS-CoV-2 infection (Healthy Control – HC, n = 8) and volunteers who recovered from mild (Mild Recovered, n = 9) and severe (Severe

Recovered, n = 7) COVID-19. **(A)** The strategy of analysis for regulatory T cells expressing IL-10, IL-17, and LAP identification and counting. The representative dot plots were derived from unstimulated samples. **(B)** Heatmap of frequency of regulatory T cells expressing IL-10, IL-17, and LAP in volunteers from the HC, Mild Recovered, and Severe Recovered groups based on flow cytometry analyses. Each row indicates the condition in which the PBMCs were submitted (shown on the right). Each column indicates a volunteer enrolled in the study. Each sequence of 4 rows indicates a marker analyzed (shown on the left). **(C.1-3)** Comparison of the frequency of regulatory T cells expressing IL-10 among Mild Recovered volunteers who had and had not experienced the following symptoms: dyspnea, sore throat, and ageusia during acute COVID-19. **(C.4-9)** Comparison of the frequency of regulatory T cells expressing IL-17 among Mild Recovered volunteers who had and had not experienced the following symptoms: dyspnea, sore throat, arthralgia, nasal obstruction, diarrhea, and ageusia during acute COVID-19. **(C.10-15)** Comparison of the frequency of regulatory T cells expressing LAP among Mild Recovered volunteers who had and had not experienced the following symptoms: myalgia, arthralgia, fatigue, diarrhea, anosmia, and ageusia during acute COVID-19.

The symbols ●, ■, and ▲ represent each sample from Mild Recovered volunteer that was unstimulated (medium condition only) or stimulated with Pool Spike CoV-2 peptides or Pool CoV-2 peptides, respectively. When appropriate, multiple comparisons were performed using one-way ANOVA and Tukey’s post hoc test or Kruskal-Wallis with Dunn’s multiple comparisons tests. The bars represent mean values, and the error bars show the standard error of the mean (SEM) for each group.

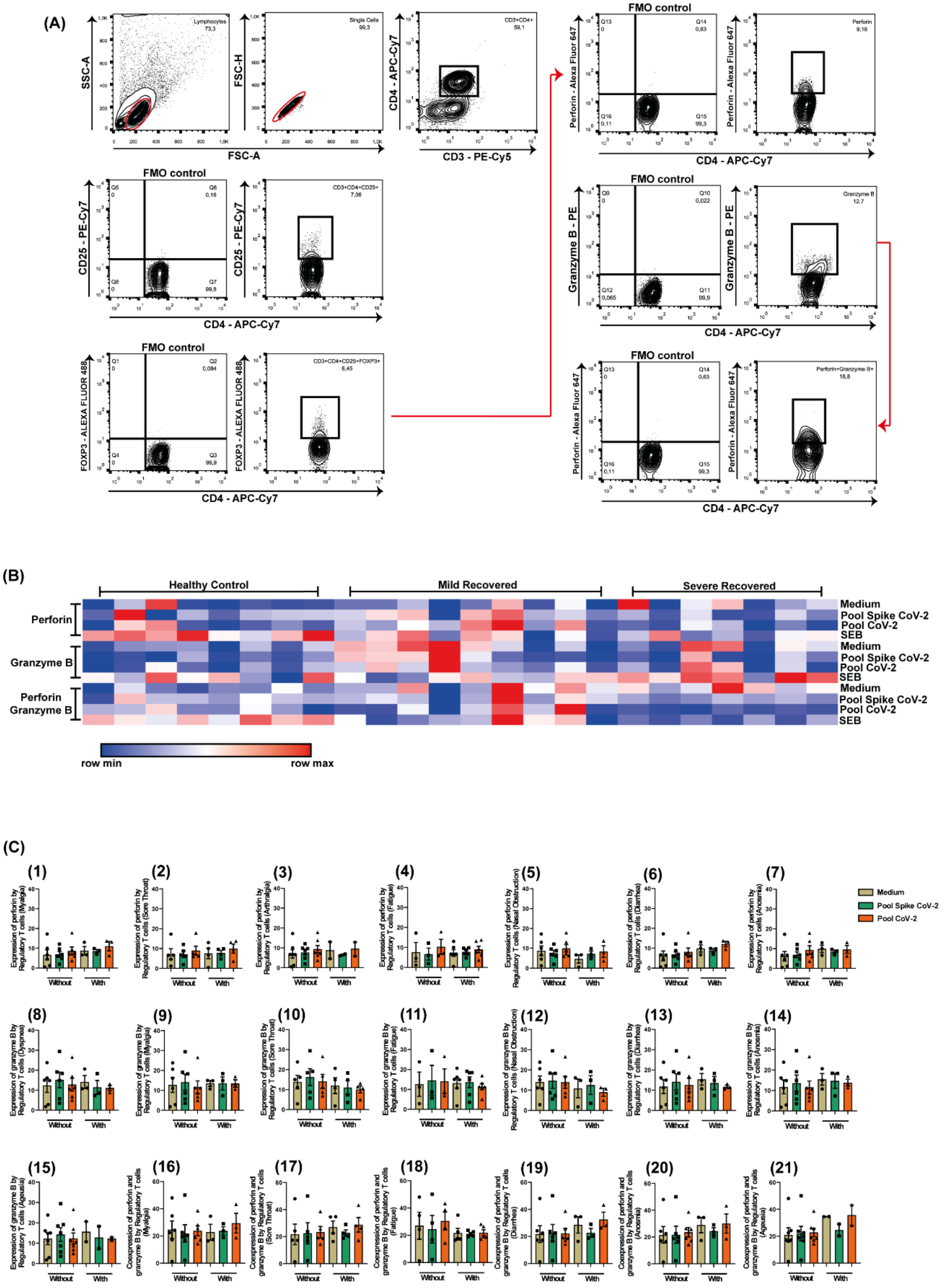

Supplementary Figure 3. **Regulatory T cells expressing perforin and granzyme B in peripheral blood from volunteers**. Peripheral blood mononuclear cells (PBMC) were collected from volunteers not previously affected by SARS-CoV-2 infection (Healthy Control - HC, n = 8) and volunteers who recovered from mild (Mild Recovered, n = 9) and severe (Severe Recovered, n = 7) COVID-19. **(A)** The strategy of analysis for regulatory T cell expressing perforin and granzyme B and coexpressing

perforin/granzyme B identification and counting. The representative dot plots were derived from unstimulated samples. **(B)** Heatmap of frequency of regulatory T cells expressing perforin, granzyme B, and coexpressing both granules in volunteers from the HC, Mild Recovered, and Severe Recovered groups based on flow cytometry analyses. Each row indicates the condition in which the PBMCs were submitted (shown on the right). Each column indicates a volunteer enrolled in the study. Each sequence of 4 rows indicates a marker analyzed (shown on the left). **(C.1-7)** Comparison of the frequency of regulatory T cells expressing perforin among Mild Recovered volunteers who had and had not experienced the following symptoms: myalgia, sore throat, arthralgia, fatigue, nasal obstruction, diarrhea, and anosmia during acute COVID-19. **(C.8-15)** Comparison of the frequency of regulatory T cells expressing granzyme B among Mild Recovered volunteers who had and had not experienced the following symptoms: dyspnea, myalgia, sore throat, fatigue, nasal obstruction, diarrhea, anosmia, and ageusia during acute COVID-19. **(C.16-21)** Comparison of the frequency of regulatory T cells coexpressing perforin and granzyme B among Mild Recovered volunteers who had and had not experienced the following symptoms: myalgia, sore throat, fatigue, diarrhea, anosmia, and ageusia during acute COVID-19.

The symbols ●, ■, and ▲ represent each sample from Mild Recovered volunteer that was unstimulated (medium condition only) or stimulated with Pool Spike CoV-2 peptides or Pool CoV-2 peptides, respectively. When appropriate, multiple comparisons were performed using one-way ANOVA and Tukey’s post hoc test or Kruskal-Wallis with Dunn’s multiple comparisons tests. The bars represent mean values, and the error bars show the standard error of the mean (SEM) for each group.

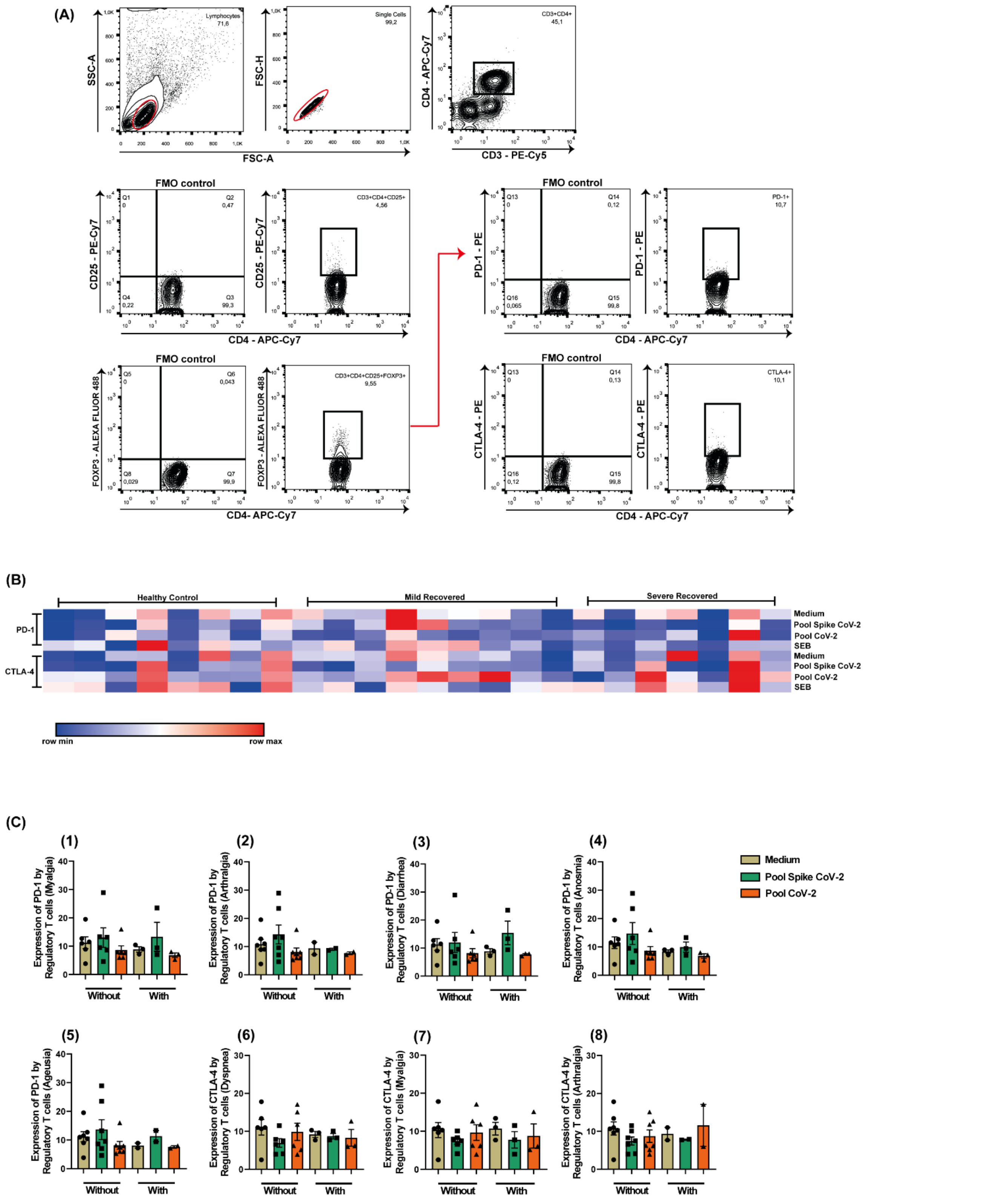

Supplementary Figure 4. **Regulatory T cells expressing PD-1 and CTLA-4 in peripheral blood from volunteers**. Peripheral blood mononuclear cells (PBMC) were collected from volunteers not previously affected by SARS-CoV-2 infection (Healthy Control - HC, n = 8) and volunteers who

recovered from mild (Mild Recovered, n = 9) and severe (Severe Recovered, n = 7) COVID-19. **(A)** The strategy of analysis for regulatory T cell expressing PD-1 and CTLA-4 identification and counting. The representative dot plots were derived from unstimulated samples. **(B)** Heatmap of frequency of regulatory T cells expressing PD-1 and CTLA-4 in volunteers from the HC, Mild Recovered, and Severe Recovered groups based on flow cytometry analyses. Each row indicates the condition in which the PBMCs were submitted (shown on the right). Each column indicates a volunteer enrolled in the study. Each sequence of 4 rows indicates a marker analyzed (shown on the left). **(C.1-5)** Comparison of the frequency of regulatory T cells expressing PD-1 among Mild Recovered volunteers who had and had not developed the following symptoms: myalgia, arthralgia, diarrhea, anosmia, and ageusia during acute COVID-19. **(C.6-8)** Comparison of the frequency of regulatory T cells expressing CTLA-4 among Mild Recovered volunteers who had and had not experienced the following symptoms: dyspnea, myalgia, and arthralgia during acute COVID-19.

The symbols ●, ■, and ▲ represent each sample from Mild Recovered volunteer that was unstimulated (medium condition only) or stimulated with Pool Spike CoV-2 peptides or Pool CoV-2 peptides, respectively. When appropriate, multiple comparisons were performed using one-way ANOVA and Tukey’s post hoc test or Kruskal-Wallis with Dunn’s multiple comparisons tests. The bars represent mean values, and the error bars show the standard error of the mean (SEM) for each group.

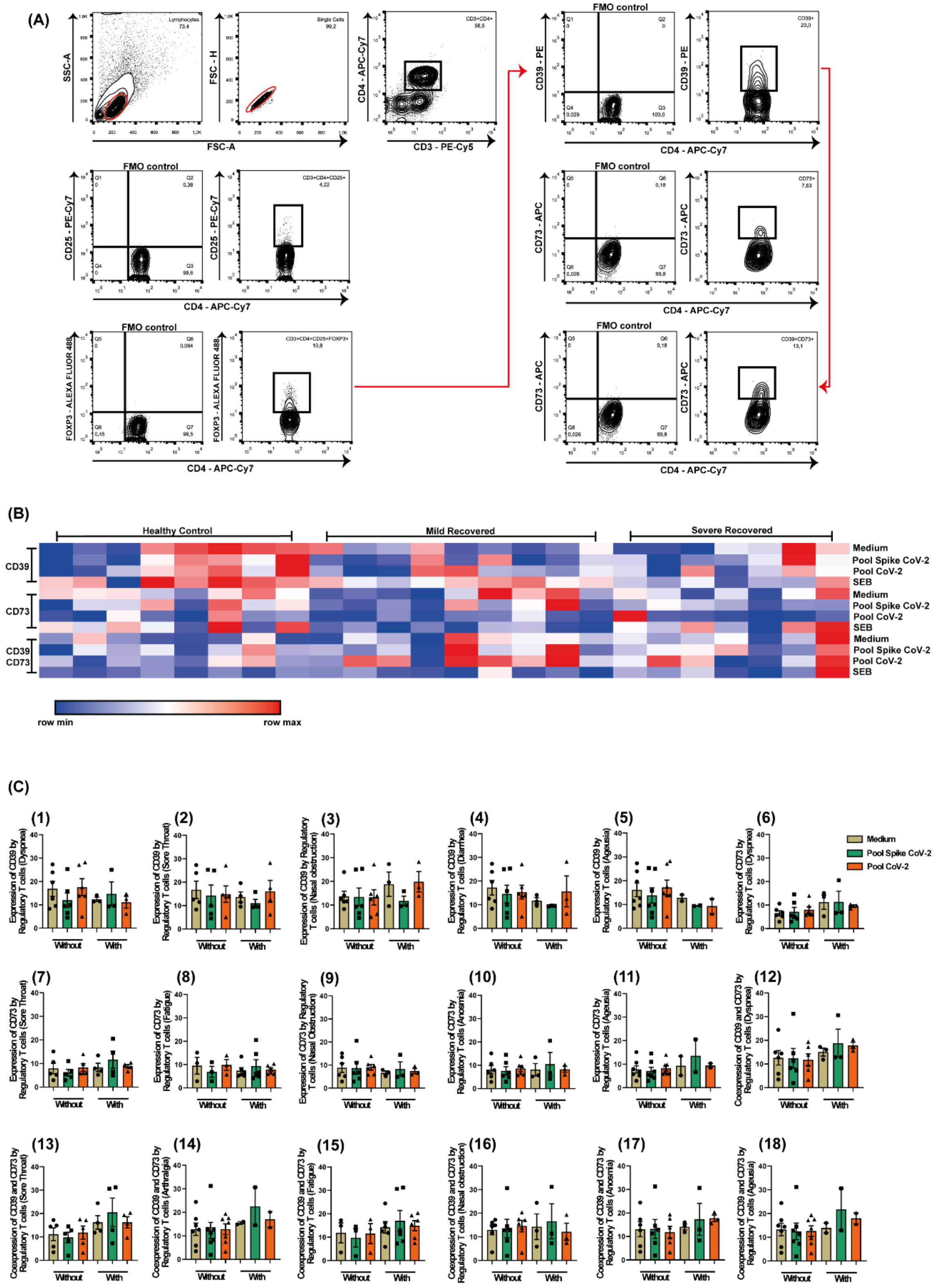

Supplementary Figure 5. **Regulatory T cells expressing CD39 and CD73 in peripheral blood from volunteers**. Peripheral blood mononuclear cells (PBMC) were collected from volunteers not previously affected by SARS-CoV-2 infection (Healthy Control - HC, n = 8) and volunteers who recovered from mild (Mild Recovered, n = 9) and severe (Severe Recovered, n = 7) COVID-19. **(A)** The strategy of analysis for regulatory T cells expressing CD39 and CD73 and coexpressing

CD39/CD73 identification and counting. The representative dot plots were derived from unstimulated samples. **(B)** Heatmap of frequency of regulatory T cells expressing CD39, CD73, and coexpressing both ectonucleotidases in volunteers from the HC, Mild Recovered, and Severe Recovered groups based on flow cytometry analyses. Each row indicates the condition in which the PBMCs were submitted (shown on the right). Each column indicates a volunteer enrolled in the study. Each sequence of 4 rows indicates a marker analyzed (shown on the left). **(C.1-5)** Comparison of the frequency of regulatory T cells expressing CD39 among Mild Recovered volunteers who had and had not experienced the following symptoms: dyspnea, sore throat, nasal obstruction, diarrhea, and ageusia during acute COVID-19. **(C.6-11)** Comparison of the frequency of regulatory T cells expressing CD73 among Mild Recovered volunteers who had and had not experienced the following symptoms: dyspnea, sore throat, fatigue, nasal obstruction, anosmia, and ageusia during acute COVID-19. **(C.12-18)** Comparison of the frequency of regulatory T cells coexpressing CD39 and CD73 among Mild Recovered volunteers who had and had not experienced the following symptoms: dyspnea, sore throat, arthralgia, fatigue, nasal obstruction, anosmia, and ageusia during acute COVID-19.

The symbols ●, ■, and ▲ represent each sample from Mild Recovered volunteer that was unstimulated (medium condition only) or stimulated with Pool Spike CoV-2 peptides or Pool CoV-2 peptides, respectively. When appropriate, multiple comparisons were performed using one-way ANOVA and Tukey’s post hoc test or Kruskal-Wallis with Dunn’s multiple comparisons tests. The bars represent mean values, and the error bars show the standard error of the mean (SEM) for each group.
